# Italian Evidence-Based Clinical Recommendations on the Appropriateness of Prescriptions and Diagnostic Tests in Pediatric Allergology: Focus on Anaphylaxis, Drug Allergy and Hymenoptera Venom Allergy

**DOI:** 10.3390/jcm15020678

**Published:** 2026-01-14

**Authors:** Valentina Fainardi, Matteo Riccò, Rachele Antignani, Simona Bellodi, Enrico Vito Buono, Mauro Calvani, Roberta Carbone, Fabio Cardinale, Elena Chiappini, Maria Angiola Crivellaro, Daniela Cunico, Massimiliano Esposito, Amelia Licari, Michele Miraglia Del Giudice, Maria Marsella, Iria Neri, Rita Nocerino, Diego Peroni, Cristina Piersantelli, Giuseppe Pingitore, Giuseppe Squazzini, Maria Angela Tosca, Carlo Caffarelli, Susanna Esposito

**Affiliations:** 1 Pediatric Clinic, Department of Medicine and Surgery, University of Parma, 43121 Parma, Italy; valentina.fainardi@unipr.it (V.F.);; 2 Servizio di Prevenzione e Sicurezza Negli Ambienti di Lavoro (SPSAL), AUSL-IRCCS Reggio Emilia, Forli, 47122 Reggio Emilia, Italy; matteo.ricco@ausl.re.it; 3 Società Italiana Medici Pediatri (SIMPE), Italy; 4 Società Italiana di Allergologia e Immunologia Pediatrica (SIAIP), Italyfabiocardinale1961@gmail.com (F.C.);; 5 UOC Pediatria, Ospedale S. Camillo-Forlanini, 00152 Roma, Italy; 6 UOC Pediatria, Ospedale Pediatrico “Giovanni XXIII”, 70126 Bari, Italy; 7 UOC Malattie Infettive, Ospedale Meyer, 50139 Firenze, Italy; 8 Società Italiana di Pediatria Preventiva e Sociale (SIPPS), Italy; 9 UOSD Allergologia Azienda Ospedale, Università di Padova, 35122 Padova, Italy; 10 Società Italiana di Medicina Legale e delle Assicurazioni (SIMLA), Italy; massimiliano.esposito@unikore.it; 11 Medicina Legale, Università Degli Studi di Enna, 94100 Enna, Italy; 12 UOC Clinica Pediatrica, IRCCS Policlinico San Matteo, 27100 Pavia, Italy; 13 Dipartimento della Donna, del Bambino e di Chirurgia Generale e Specialistica, Università della Campania Luigi Vanvitelli, 80138 Napoli, Italy; 14 Società Italiana Gestori del Rischio in Sanità (SIGeRiS), Italy; m.marsella79@gmail.com; 15 UOC di Pediatria Azienda Ospedaliera di Rilievo Nazionale San Giuseppe Moscati di Avellino, 83100 Avellino, Italy; 16 Società Italiana di Dermatologia Pediatrica (SIDeRP), Italy; 17 UOC Clinica Dermatologica, IRCCS Azienda Ospedaliera Universitaria di Bologna, 40138 Bologna, Italy; 18 Società Italiana di Pediatria Infermieristica (SIPINF), Italy; rita.nocerino@unina.it; 19 Clinica Pediatrica, Università di Pisa, 56126 Pisa, Italy; 20 Società Italiana delle Cure Primarie Pediatriche (SICuPP), Italy; 21 Società Italiana di Malattie Respiratorie Infantili (SIMRI), Italy; giuseppe.pingitore@gmail.com; 22 Centro di Allergologia, IRCCS Giannina Gaslini, 16147 Genova, Italy

**Keywords:** pediatric allergology, evidence-based recommendations, anaphylaxis, drug allergy, hymenoptera venom allergy

## Abstract

**Background/Objectives:** Evidence-based recommendations are vital in healthcare to standardize care, reduce variability, and improve patient outcomes. In children, anaphylaxis, allergy to antibiotics, and hymenoptera venom allergy are among the commonest reasons for allergological evaluation. This work was intended to optimize the prescriptions for allergological evaluation and for the related diagnostic tests with the aim of improving the management of children with allergic diseases and promoting resource efficiency. **Methods:** A systematic literature review of the literature was performed to formulate recommendations on the diagnostic management of children with anaphylaxis, drug allergy, and hymenoptera venom allergy. **Results:** Effective management of anaphylaxis involves rapid assessment and specialist follow-up to identify triggers, prevent recurrence, and ensure patients and caregivers are educated and equipped with an adrenaline auto-injector. Integrating skin testing, specific serological assays, and oral provocation tests into the diagnostic process for children with suspected beta-lactam allergy enhances diagnostic accuracy and minimizes unnecessary avoidance of first-line antibiotics. Children and adolescents with systemic reactions to hymenopteran stings should be referred to an allergy specialist for diagnosis, risk assessment, management education, and adrenaline prescription. **Conclusions:** These recommendations may enhance care quality, minimize inappropriate prescriptions, and support standardized methods of diagnosis of allergological diseases in children.

## 1. Introduction

The appropriateness of prescriptions for clinical and instrumental tests plays a role in managing waiting lists and provides consistency and equity in access to care. Conversely, inappropriateness results in waste of time and resources for patients and their families and in unnecessary burden for specialists and the healthcare system. For this reason, the production of clear, shared recommendations based on the best available scientific evidence can be instrumental for improving the quality of care and optimizing the use of resources, ultimately contributing to the sustainability of the National Health Service [1]. Due to the significant burden in pediatric age subjects, Good Clinical Practice Recommendations (GCPR) may be of substantial significance in the specific setting of pediatric allergology, particularly when dealing with conditions associated with potentially life-threatening conditions such as anaphylaxis, including the specific subtheme of hymenoptera venom allergy (HVA), and drug allergy (DA) [2,3,4,5]. The global prevalence of anaphylaxis in children ranges from 0.04% to 1.8% depending on countries [6]. Considering the emergency department (ED) visits, the median hospitalization rate is 3.5% [7]. Anaphylaxis caused by hymenoptera venom affects between 0.3% and 8.9% of individuals who are stung. Nevertheless, it remains the leading cause of anaphylaxis and is responsible for 20% of all fatal cases of anaphylaxis [8,9,10]. In children the prevalence of drug hypersensitivity reactions ranges from 1% to 10.2% and 4–9% of these reported cases are confirmed as true allergic [11].

The challenging task of designing up-to-date and effective GPCS, four main principles must be preliminarily fulfilled: (1) clarity in the definition of each attribute; (2) compatibility of each attribute and its definition with professional usage; (3) clear rationales and justifications for the selection of each attribute, and the (4) sensitivity to practical issues in using the attribute to assess actual sets of practice guidelines (i.e., “accessibility”). In turn, when properly defined, aforementioned attributes should guarantee validity (i.e., when followed, GCPR lead to the projected health and costs outcomes), reliability/reproducibility, clinical applicability, clinical flexibility, clarity, and—finally yet importantly—multidisciplinarity of all newly designed GCPR.

The aim of the work was the creation of shared recommendations for Italian pediatricians based on the best available evidence to reduce variability of prescriptions, harmonize medical practices at a national level, improve equity in access to health services, on the following themes: diagnostic management of anaphylaxis, DA, and HVA among children and adolescents.

## 2. Methods

### 2.1. Working Group and Panel of Experts

The present document is the result of the joint work of a multidisciplinary panel, which includes representatives of pediatrics scientific societies (Italian Society of Pediatricians [Società Italiana Medici Pediatri, in Italian; SIMPE], Italian Society of Pediatrics [Società Italiana di Pediatria, in Italian; SIP], Italian Society of Pediatric Allergology and Immunology [Società Italiana di Allergologia e Immunologia Pediatrica, in Italian; SIAIP], Italian Society for Childhood Respiratory Diseases [Società Italiana per le Malattie Respiratorie Infantili, in Italian, SIMRI], Italian Society of Pediatric Emergency Medicine [Società Italiana di Medicina di Emergenza e Urgenza Pediatrica, in Italian; SIMEUP], Italian Society of Pediatric Primary Care [Società Italiana delle Cure Primarie Pediatriche, in Italian; SICuPP]); representatives of scientific societies of specialists involved in the care of allergic children (Italian Society of Pediatric Dermatology [Società Italiana di Dermatologia Pediatrica, in Italian; SIDerP], Italian Association of Local and Hospital Allergists and Immunologists [Associazione Allergologi Immunologi Italiani Territoriali e Ospedalieri, AAITO]), as well as experts in legal medicine from the Legal and Insurance Medicine (Società Italiana di Medicina Legale e delle Assicurazioni, in Italian; SIMLA), and in telemedicine from Italian Telemedicine Society, (Società Italiana di Telemedicina, in Italian; SIT).

In order to guarantee the multidisciplinarity of GCPR on the topics of anaphylaxis, HVA, and DA, a multi-professional working group was designed and included pediatric allergologists, general pediatricians, general practitioners, clinical immunologists, otolaryngologists, dermatologists, clinical pharmacologists, psychologists, and pediatric nurses. The group also had methodologists and representatives of patient associations to respond to the concrete needs of the families. This study was part of a broader initiative aimed at producing evidence-based GCPR for prescriptions in pediatric patients with allergic diseases convened under the Italian Superior Institute of Health (Istituto Superiore di Sanità, in Italian; ISS).

### 2.2. Formulation of Clinical Questions

As a preliminary step, research questions on the topics of diagnostic management of anaphylaxis, DA, and HVA among children and adolescents were discussed and then developed by the working group according to the model population/patient (P), intervention (I), comparator (C), outcome (O), or PICO [12,13]. Each member of the working group was invited to share a total of five PICOs for each assessed topic for a total of 15 PICOs. All PICOs were then gathered and scored by experts through a LIKERT scale (0–3 not agreeing, 4–6 moderately agreeing, 7–9 strongly agreeing): only PICOs with an average score > 7 were eventually included into the subsequent analyses.

### 2.3. Systematic Review

To answer the research questions eventually approved by participating experts, a series of systematic review of the literature was performed. Three different databases (Medline, Embase, and Cochrane) were searched, exploring specific keywords such as “allergy,” “atopy,” “allergic disease,” and additional keywords for each considered condition (the complete search strings are available in the Table A1). Inclusion criteria were the following ones:

- Design of the source studies: only randomized controlled trials (RCTs), observational cohort studies with a comparator, case–control studies were included; regarding secondary sources, systematic reviews with and without meta-analyses and guidelines were initially retrieved and then assessed for obtaining additional entries through a snowball approach. Conversely, case reports, letters to the editor, brief reports, and conference abstracts were excluded;
- Age groups, only studies reporting on subjects aged 0–18 years were considered;
- Language and timeframe: the search was arbitrarily limited to results in English, published between January 2015 and January 2025.

According to the Preferred Reporting Items for Systematic Reviews and Meta-Analyses (PRISMA) statement [12,13], suitable articles were initially pooled together. After the removal of duplicates, two researchers conducted title and abstract screening independently and blindly. Articles fulfilling inclusion criteria were then similarly blindly reviewed by two researchers. Conflicts in each state of the systematic review were resolved through discussion with a third researcher (see Prisma Checklist as Supplementary Materials Table S1).

Risk of bias of observational studies was assessed by means of the Newcastle–Ottawa scale (NOS, potential range from 0 to 9 points). NOS has been extensively recommended by Cochrane Collaboration for the qualitative assessment of source studies to be implemented into systematic reviews [14]. The NOS scale consists of four domains of risk of bias assessment: (1) selection bias; (2) performance bias; (3) detection bias; and (4) information bias. All of them have been specifically tailored for case–control and cohort studies, while no specific recommendation was originally identified for cross-sectional studies. However, as cross-sectional studies and cohort studies share a similar structure, except for the timing of the measurement for exposures and outcomes (i.e., at the same time, or cross-sectionally, for cross-sectional studies; after the exposure for cohort studies) [15] we opted to apply the cohort studies scale also to cross-sectional ones. According to the current indications and the study protocols, two investigators independently rated all suitable articles and provided a summary of their potential shortcomings. Potential disagreements were primarily resolved by consensus between the two reviewers; input from a third investigator was requested and obtained when consensus was not possible. Predefined thresholds were defined as follows: 0–3 low quality, 4–6 moderate quality, 7–9 high quality. In order to gather the largest available sample of studies, we tentatively included into the analyses all studies fulfilling inclusion criteria despite their eventual quality.

For RCT, ROB tool from the National Toxicology Program (NTP)’s Office of Health Assessment and Translation (OHAT) (now the Health Assessment and Translation (HAT) group) was implemented [16,17]. OHAT ROB provides a 4-point scale rating (from “definitely low”, “probably low”, and “probably high” to “definitely high”) on the following potential sources of bias: participant selection (D1), confounding factors (D2), attrition/exclusion (D3), detection (D4), and selective reporting (D5), as well as other sources of bias (D6). OHAT ROB was prioritized over other instruments for ROB assessment, as it does not provide an overall rating for each study, nor does it require that studies affected by a certain degree of ROB be removed from the pooled analyses [17].

### 2.4. Data Extraction

Articles meeting all inclusion criteria were then selected for data extraction, including title, authors, journal, and year of publication, target population (by disease and age), type of intervention and comparator, and results. Where available, numerical data were extracted for possible meta-analysis; if unavailable, results were reported narratively.

### 2.5. Meta-Analysis

When at least five studies evaluated the same type of intervention in the same population and reported the same outcome, a meta-analysis was conducted. For dichotomous data, if derived from randomized controlled trials, Relative Risk with a 95% confidence interval was used; for observational studies, Odds Ratio with a 95% confidence interval was used. For continuous variables, the mean difference or standardized mean difference was used if different scores were used to evaluate the same outcome. These analyses were conducted when the mean, standard deviation, and sample size of each group were available. If the mean and standard deviation were not directly available but other data, such as the confidence interval, were available, the standard deviation was imputed from the available data. If data were not available, an email was sent to the corresponding author requesting the data to include the study in the meta-analysis.

The meta-analysis was conducted by means of R (version 4.3.1) [18] and Rstudio (version 2023.06.0 Build 421; Rstudio, PBC; Boston, MA, USA) software by means of the packages meta (version 7.0) and fmsb (version 0.7.5).

### 2.6. GRADE and GRADE Framework

To assess the quality of the evidence found, the GRADE (Grading of Recommendations Assessment, Development, and Evaluation) method was applied. For each PICO, the different outcomes considered were evaluated by dividing them into subgroups based on the type of intervention. For each outcome, the quality of the evidence found was assessed, considering the type of study included, the evaluation of biases, the lack of reproducibility of results, the lack of generalizability, imprecision, and the presence of any additional considerations related to possible publication biases. For each intervention for each PICO, different aspects were evaluated: the magnitude of the problem, the desired effect, the undesired effect, the certainty of the evidence, the value of the studies found, the benefit-risk balance, the resources required, the certainty of the evidence for the resources required, the cost–benefit balance, equity, acceptability, and sustainability.

### 2.7. Consensus Panel for the Strength of Recommendations

To reach a consensus on the strength of the recommendations, a consensus was conducted among the expert panel through a survey. Once the recommendations were formulated, a survey was sent to each member of the panel. Using a Likert scale from 0 to 9, each expert was asked to express their opinion on the strength of the recommendation (0–3 low strength, 4–6 moderate, 7–9 strong) and on sustainability (0–3 not sustainable, 4–6 probably sustainable, 7–9 certainly sustainable). The average scores given by each expert were then used to define the strength of the recommendations. In case of highly contradictory responses, the survey results were discussed among the expert panel.

The development process of the recommendations was in accordance with the standards defined by the document “Methodological guidelines for drafting recommendations for good clinical and care practices”.

## 3. Results

The risk of bias assessment of each study has been reported in Table A2 and Table A3, while the detailed description of the included studies is reported in Table A4, Table A5, Table A6, Table A7, Table A8, Table A9 and Table A10. The quality of evidence, evaluated with GRADE methods, within the GRADE Framework, is summarized by the end of each of the following sections.

### 3.1. Anaphylaxis

#### 3.1.1. Summary of Literature Search

**Figure 1:**
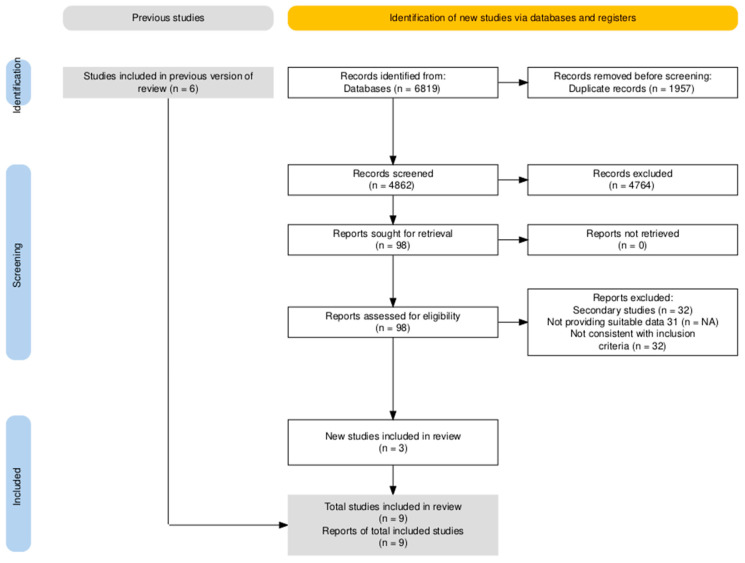
PRISMA flowchart for anaphylaxis of included studies.

The research on three databases (Embase, Medline, Cochrane) identified a total of 6819 articles. Study selection process is presented in the PRISMA flow diagram reported in Figure 1.

Briefly, after the removal of duplicates across included databases (n = 1957; 28.7% of initial sample), remaining 4862 studies were title and abstract screened. Most of the articles were considered as not consistent with the inclusion criteria: 4764 articles (69.9% of the original sample) were therefore removed from the analyses, with 98 studies (1.4%) considered as suitable for being full-text screened. In accord with the expert opinion, 6 additional articles were then included outside the original database search, bringing the total number of assessed entries to 104. Nine articles were ultimately included in the final analyses: five of them were observational studies, and 4 were RCTs (Table A3, Table A4 and Table A5).

#### 3.1.2. Clinical Questions for Anaphylaxis

##### PICO 1

For children and adolescents suspected of having anaphylaxis (P), is a deferred-urgency allergy evaluation recommended in order to identify the triggering factor, prescribe an adrenaline auto-injector (AAI) with appropriate training in its use and provide strategies for preventing and managing recurrences, (I), rather than routine follow-up with a primary care pediatrician (C), thereby reducing the risk of recurrence and emergency department visits (O)?

According to the specific P.I.C.O. question, the main outcomes considered included:

- The diagnostic accuracy of allergy tests in identifying the causal agent and initiating a specific management pathway.
- The risk of recurrent anaphylactic episodes.

Evidence (Quantitative analysis): Due to the heterogenous design of retrieved studies, both in terms of targeted population and included outcomes, no quantitative summary of evidence was ultimately performed by means of a meta-analytical approach.

Evidence (Narrative report): The World Allergy Organization (WAO) Anaphylaxis Committee defines anaphylaxis as a severe, systemic hypersensitivity reaction which usually develops rapidly and may be fatal. The most critical presentations involve a life-threatening compromise of the airway, breathing, and/or circulation. This can occur even when there are no typical skin symptoms or circulatory shock [2]. The first-line treatment for anaphylaxis is intramuscular (IM) adrenaline, which is administered into the anterolateral thigh. If the clinical response is inadequate, a second dose may be administered after 5–10 min; most reactions resolve following one or two injections [19,20].

The 2021 update of the Resuscitation Council United Kingdom (RCUK) guidelines highlighted persistent deficiencies in the recognition, treatment and follow-up of anaphylaxis. IM adrenaline was not administered in approximately 50% of reported cases, primarily due to failure to recognize anaphylaxis [21].

Diagnosis can be particularly challenging in children due to the variable and sometimes non-specific clinical presentation, ranging from mild to severe, potentially life-threatening reactions [22]. Among patients with anaphylaxis of unknown etiology, only 56% were referred to an allergologist, who subsequently identified the causative agent in 38% of cases [17].

Referral to a tertiary allergy centre and structured long-term follow-up are essential for identifying individual risk factors, implementing allergen avoidance strategies, preventing recurrences and providing targeted patient education. Furthermore, referral to an allergologist is associated with higher rates of AAI prescription [23].

This approach is particularly important for patients deemed to be at increased risk of recurrence, including those with a history of severe reactions, known triggers (e.g., peanuts or tree nuts), or coexisting asthma [22,24].

Both the WAO and the RCUK guidelines recommend the prompt and specialized management of anaphylaxis. A specialist evaluation by a qualified allergologist is required to identify the trigger, prescribe an AAI, provide training on its correct use, and establish a comprehensive prevention plan [2,21].

Education on the adrenaline self-administration is a key component of anaphylaxis management. In a randomized controlled trial, Umasunthar et al. demonstrated a high rate of correct AAI use six weeks after training, with correct technique still being retained after one year without further training. However, when a different device model was provided without additional training, success rates decreased significantly [25].

There are several adrenaline delivery devices available. The most common of these are auto-injectors, which are single-use, pre-dosed devices that can be stored at room temperature and used safely by non-medical individuals. In settings where auto-injectors are unavailable, prefilled syringes are an acceptable alternative [26].

Structured education and hands-on training in the auto-injector use improve patients’ quality of life by reducing anxiety and increasing confidence in managing future reactions [27]. Evidence from epidemiological studies, animal models, and small-scale human studies suggests that the timely use of AAI may prevent fatal outcomes [28].

However, various concerns identified by parents may prevent or delay the utilization of AIA, including reluctance to carry the device, apprehension regarding needles, and insufficient training [29]. Recently, epinephrine nasal spray (neffy) was approved as the first needle-free epinephrine option for the treatment of anaphylaxis for adults and children >15 kg [30,31]. The diffusion of this device in the community of patients may have a positive impact on the management of anaphylaxis, particularly in pediatric age, overcoming the barrier of needle-phobia and promoting early administration. Despite clear recommendations, anaphylaxis remains under-recognized and under-treated, with patients not being referred for specialist follow-up care as often as they should be. Greater specialist involvement is required to improve clinical outcomes and reduce recurrence risk [2,21].

The ANA-PED study revealed persistent shortcomings in the management of pediatric anaphylaxis in emergency departments. Notably, only 17% of children were administered adrenaline during their visit, while 28.1% were prescribed an AAI upon discharge. Furthermore, only 57.1% were referred for specialist allergy follow-up [32].

**Recommendation** (Table 1): Due to the moderate quality of evidence, a consensus-based recommendation rather than an evidence based was framed. The management of anaphylaxis requires both prompt assessment of the acute episode and a long-term allergy evaluation. Specialist consultation is essential to identify the causative triggers, implement effective allergen avoidance strategies, provide education to patients and caregivers on prevention of future reactions and prescribe AAI.

##### PICO 2

For children and adolescents suspected of having anaphylaxis (P), is measuring of serum tryptase (from 30 min to 4 h after onset of the reaction) and baseline tryptase (after complete resolution of symptoms, at least 24 h later) (I) recommended over clinical follow-up alone (C), to facilitate diagnosis, stratify risk (e.g., identify underlying conditions such as systemic mastocytosis or predisposition to more severe reactions) and improve long-term management (preventing new episodes)? (O)

Evidence (Quantitative analysis): Due to the heterogenous design of retrieved studies, both in terms of targeted population and included outcomes, no quantitative summary of evidence was ultimately performed by means of a meta-analytical approach.

Evidence (Narrative report): In a study involving 965 children with anaphylaxis, serum tryptase levels were measured within 2 h of symptom onset in 203 cases, and 19.2% of these showed elevated levels (≥11.4 μg/L). This finding was significantly associated with severe reactions. The same study compared tryptase concentrations during the acute phase and after recovery. It demonstrated a mean difference of 6.3 μg/L, indicating that tryptase levels in the blood were significantly higher during the acute episode than at baseline. These results reinforce the use of tryptase as a mast cell activation biomarker and confirm its value in diagnosing atypical or uncertain cases of anaphylaxis [33]. Conversely, the study by Cavkaytar et al. [34], which involved 345 children suspected of having drug-induced anaphylaxis, found that baseline serum tryptase levels were similar in those with confirmed drug hypersensitivity (n = 106) and in healthy controls. While these contrasting findings suggest that serum tryptase is a poor predictive biomarker, the 2020 WAO anaphylaxis guidelines [2] nevertheless recommend measuring serum tryptase levels in cases of anaphylaxis. A significant elevation in serum tryptase levels can support the diagnosis, particularly in cases with atypical presentations or absent cutaneous manifestations. Comparing serum tryptase levels immediately after an anaphylactic episode with baseline levels may help to identify predisposing conditions, such as systemic mastocytosis or other mast cell disorders, which increase the risk of severe or treatment-resistant reactions [35]. Children with cutaneous mastocytosis have been shown to exhibit elevated baseline serum tryptase levels and may be at increased risk of anaphylaxis, especially when skin involvement is extensive [36]. Furthermore, elevated levels of serum tryptase are found in hereditary α-tryptasemia (HαT), a genetic trait caused by increased α-tryptase-encoding Tryptase-α/β1 (TPSAB1) copy number. This condition is a known genetic risk factor for mast cell activation and severe anaphylaxis episodes [37].

However, tryptase levels should not be used as the sole criterion for diagnosis, but rather as part of a comprehensive allergological assessment that takes into account clinical findings and the patient’s medical history [36].

**Recommendation** (Table 2): Due to the moderate quality of evidence, a consensus-based recommendation rather than an evidence based was framed. Measuring serum tryptase levels during an anaphylactic episode and at baseline is particularly valuable in cases where the diagnosis of anaphylaxis is uncertain or atypical. It can help to confirm a diagnosis of anaphylaxis and to identify underlying conditions, such as systemic mastocytosis.

##### PICO 3

In children and adolescents with suspected food allergy–related anaphylaxis (P), is performing skin prick testing (SPT) with both commercial extracts and fresh foods (I) recommended over clinical follow-up alone (C) to prevent new episodes, improve diagnostic accuracy, avoid unnecessary elimination diets, and enhance quality of life (O)?

Evidence (Quantitative analysis): Due to the heterogenous design of retrieved studies, both in terms of targeted population and included outcomes, no quantitative summary of evidence was ultimately performed by means of a meta-analytical approach.

Evidence (Narrative report): SPT is recommended for identifying allergens responsible for anaphylactic reactions [2]. However, the current guidelines emphasize that SPT results should always be interpreted alongside a detailed clinical history, since Immunoglobulin E (IgE)-mediated sensitization does not necessarily indicate a clinically significant allergic response.

Food-induced anaphylaxis presents with variable clinical features, and SPT plays a central role in identifying common triggers such as peanuts, tree nuts, and shellfish [38]. Although SPT is generally considered safe, the WAO guidelines [2] recommend that testing is performed in settings equipped with emergency facilities and staffed by personnel trained to recognize and manage anaphylaxis and other adverse reactions. Patients should be monitored for an appropriate observation period following testing to detect potential systemic responses. When SPT results are inconclusive or inconsistent with the clinical history, additional diagnostic investigations, such as serum-specific IgE measurement or an oral food challenge, may be required to establish a definitive diagnosis of anaphylaxis. Diagnostic assessment of food allergy and anaphylaxis should always begin with a comprehensive clinical history, followed by targeted testing, including SPT, measurement of specific IgE, and, in selected cases, the basophil activation test (BAT) [39,40]. Specific IgE may be useful when skin testing is unavailable, when standardized extracts do not exist, or when longitudinal monitoring is needed as children outgrow food allergies. However, The oral food challenge remains the gold standard for diagnostic confirmation [38], but it should only be performed in specialized clinical settings due to the potential risk of inducing systemic reactions [3,40]. Children with confirmed food allergies should undergo regular re-evaluation, using repeat allergy testing and/or oral food challenges, to assess whether they have developed a natural tolerance and to support the safe reintroduction of previously excluded foods [3]. A recent systematic review and meta-analysis of risk factors for severe allergic reactions to foods demonstrated that, while traditional diagnostic tools such as SPT and specific IgE testing are valuable for identifying sensitization and supporting diagnosis, they have no predictive value for assessing the severity of allergic reactions [41].

**Recommendation** (Table 3): Due to the moderate quality of evidence, a consensus-based recommendation rather than an evidence based was framed. SPT represents the first-step diagnostic tool for identifying allergen sensitization in patients suspected of having food-induced anaphylaxis. The oral food challenge remains the gold standard for definitive diagnosis.

##### PICO 4

In children and adolescents suspected of having anaphylaxis (P), is it recommended that specific IgE is measured by means of component-resolved diagnostics (CRD) (I), rather than relying solely on clinical follow-up (C), in order to identify individual risk profiles, personalize prevention strategies, and prevent new episodes (O)?

Evidence (Quantitative analysis): Due to the heterogenous design of retrieved studies, both in terms of targeted population and included outcomes, no quantitative summary of evidence was ultimately performed by means of a meta-analytical approach.

Evidence (Narrative report): CRD enables the identification and quantification of IgE antibodies directed against individual allergenic protein components, offering greater molecular specificity than conventional assays, which measure IgE reactivity to whole allergen extracts [42]. CRD is particularly valuable in complex clinical contexts, such as idiopathic anaphylaxis, where it may help detect causal allergens that are not easily identified through traditional testing. It also facilitates the distinction between true clinical allergy and cross-reactivity, for example, in cases involving hazelnut or peanut allergy [43]. CRD should be regarded as a complementary diagnostic tool rather than a standalone method. Its results should always be interpreted within the framework of a comprehensive clinical assessment [2]. However, CRD has certain limitations, such as lower sensitivity compared to extract-based diagnostics and variability in performance depending on the studied population and the routes of sensitization [42]. Consequently, CRD should be employed as an adjunct to conventional diagnostics, with its clinical relevance evaluated on an individual basis. Furthermore, the lack of standardized diagnostic thresholds and the limited availability of CRD in certain clinical settings currently restricts its wider implementation. SPTs and specific serum IgE measurements are unreliable indicators of the severity of a reaction or the risk of anaphylaxis during oral food challenges. Conversely, allergenic components identified through CRD can provide information about the risk of severe reactions, especially when combined with other clinical markers. For instance, monosensitization to Arachis hypogaea (Ara h) 8 in peanut allergy is linked to a reduced likelihood of anaphylaxis and frequently indicates pollen–food allergy syndrome (PFAS). In contrast, sensitization to proteins such as Prunus persica (Pru p) 3 (peach) and 2S albumins in tree nut allergies has been linked to an increased risk of systemic reactions [41]. The 2023 European Academy of Allergy and Clinical Immunology (EAACI) Guidelines support the use of CRD in the evaluation of sensitization to specific foods, including peanuts, hazelnuts and cashews. Measurement of IgE to allergen components such as Ara h 2, Corylus avellana (Cor a) 14, and Anacardium occidentale (Ana o) 3 can enhance diagnostic accuracy, particularly in patients with concomitant pollen sensitization [3]. In this context, the study by Kukkonen et al. provides further evidence supporting the use of CRD in peanut allergy. In a double-blind, placebo-controlled food challenge involving children and adolescents, the authors found that sensitization to Ara h 2 and Ara h 6 was strongly associated with moderate to severe allergic reactions. Moreover, the combined measurement of IgE to Ara h 2 and Ara h 6 accurately identified all individuals who experienced severe reactions at low allergen doses. By contrast, IgE to Ara h 8 did not correlate with clinically relevant reactions, thus confirming its limited prognostic value in severe peanut allergy [44]. Similarly, other research has shown that although CRD improves the differentiation between allergic and non-allergic children to peanuts, IgE levels to individual components, including Ara h 2 and Ara h 8, do not consistently correlate with reaction severity or eliciting dose during oral food challenges. These findings underscore the current limitations of CRD as a prognostic tool [45]. These findings suggest that the detection of specific IgE, particularly to Ara h 6, may provide significant prognostic information, potentially reducing the need for oral food challenges in selected patients and contributing to risk stratification [44].

**Recommendation** (Table 4): Due to the low to moderate quality of evidence, a consensus-based recommendation rather than an evidence based was framed When used by allergologists in an appropriate clinical context, CRD is an advanced tool that can improve the management of food-induced anaphylaxis. Evidence supports its role in evaluating the risk of severe allergic reactions in patients with specific sensitization profiles and allergen types.

### 3.2. Drug Allergy

#### 3.2.1. Summary of Literature Research

**Figure 2:**
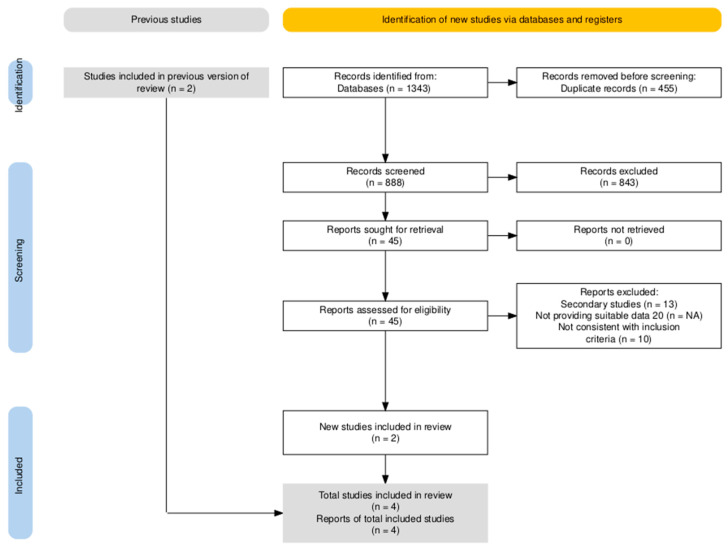
PRISMA flowchart for included studies on drug allergy.

Study selection process is presented in the PRISMA flow diagram reported in Figure 2.

The systematic search across three databases (Embase, Medline, Cochrane) identified 1343 entries: after the removal of duplicates and articles not suitable with the aims of the literature search (n = 1298, 96.6%), 45 studies were full-text screened (3.4%): two additional articles not included in the search result were added based on expert opinion. A total of 13 articles were therefore identified. Of them, only four were included into the final analyses as observational studies: no RCTs were retrieved, and all of the remaining articles were secondary studies, i.e., meta-analyses, literature reviews, and guidelines/recommendations.

The studies included into the final analyses are reported and described in Table A7 and Table A8.

The outcomes considered, according to the P.I.C.O. framework, mainly concerned:

- The diagnostic accuracy of allergy tests to identify the causal agent;
- The inappropriate use of antibiotics.

#### 3.2.2. Clinical Questions on Drug Allergy

##### PICO 1

In children and adolescents suspected of having a beta-lactam antibiotic allergy (P), does using skin tests, oral provocation tests (OPT) and specific serological assays (I) improve diagnostic accuracy, reduce misdiagnoses and decrease the use of less effective alternative treatments (O), compared with a diagnosis based solely on clinical history (C)?

Evidence (Quantitative analysis): Due to the heterogenous design of retrieved studies, both in terms of targeted population and included outcomes, no quantitative summary of evidence was ultimately performed by means of a meta-analytical approach.

Evidence (Narrative report). The diagnosis of a beta-lactam allergy in children and adolescents should not be based solely on the patient’s medical history, as this approach carries a high risk of overdiagnosis and may lead to the unnecessary avoidance of first-line antibiotics [4,46,47,48]. Incorporating structured diagnostic tools, such as skin tests, specific IgE assays, and OPT, has been shown to significantly improve diagnostic accuracy, reduce false-positive results, and limit the use of less effective alternative antibiotics [4,46,47,48]. There have been conflicting results reported regarding OPT. Goh et al. [49] found that the majority of children undergoing OPT experienced negative results. Conversely, other studies [50,51] have highlighted the high safety and diagnostic yield of OPT, particularly in children presenting with mild or delayed cutaneous reactions. Although skin tests are less definitive, they are still useful for identifying children who are at a higher risk of immediate hypersensitivity. However, they are not very sensitive or specific, which reinforces the need to proceed with oral provocation testing [52]. The combined use of detailed clinical history, skin testing, and OPT markedly improves overall diagnostic reliability. In selected cases, repeating tests may be warranted when initial results are inconclusive [46,53]. By accurately identifying patients with genuine allergies, clinicians can safely prescribe first-line beta-lactam antibiotics to most children who are not allergic. This supports a more rational and evidence-based approach to antibiotic stewardship [54,55].

**Recommendation** (Table 5): Due to the moderate quality of evidence, a consensus-based recommendation rather than an evidence based was framed. Integrating skin testing, specific serological assays and oral OPT into the diagnostic pathway for children and adolescents suspected of having a beta-lactam allergy improves diagnostic accuracy and reduces the unnecessary avoidance of effective first-line antibiotics.

##### PICO 2

In children and adolescents suspected of having an antibiotic allergy (P), does using a targeted diagnostic pathway before prescribing alternative antibiotics (I) reduce the inappropriate use of second-line agents (O), compared with empirically prescribing unrelated antibiotics (C)?

Evidence (Quantitative analysis): Due to the heterogenous design of retrieved studies, both in terms of targeted population and included outcomes, no quantitative summary of evidence was ultimately performed by means of a meta-analytical approach.

Evidence (Narrative report): In children and adolescents with suspected beta-lactam allergy, a targeted diagnostic pathway may substantially reduce the inappropriate use of second-line antibiotics, thereby improve patient safety and support antimicrobial stewardship [46,48,50]. Many reported penicillin allergies are not true IgE-mediated reactions but are often based on historical information or non-allergic adverse effects. Studies suggest that up to 90% of people who think they are allergic to penicillin can safely take the drug following appropriate allergy re-evaluation [47,56]. Consequently, empirical avoidance of beta-lactam antibiotics often results in unnecessary use of broader-spectrum or second-line agents, increasing the risk of adverse events and contributing to the development of antimicrobial resistance [52]. The implementation of a structured diagnostic algorithm, including skin testing and stepwise OPT, may enable an accurate identification of true allergic reactions. For instance, one study showed that direct OPT is both safe and effective in diagnosing beta-lactam allergy in low-risk children with mild skin symptoms [54]. Furthermore, negative skin test results can facilitate the safe reintroduction of beta-lactam antibiotics, thereby reducing the requirement for alternative second-line alternatives [4]. Guidelines from the Spanish Society of Infectious Diseases and Clinical Microbiology (SEIMC), the EAACI Position Paper, and the SIAIP Position Paper all emphasize the importance of confirming suspected antibiotic allergies using the correct diagnostic procedures before prescribing alternative therapies. This strategy ensures more effective treatments and is aligned with the principles of antimicrobial stewardship, helping to preserve the efficacy of first-line antibiotics [4,47,48,57].

**Recommendation** (Table 6): Due to the very low quality of evidence, a consensus-based recommendation rather than an evidence based was framed A targeted diagnostic approach for suspected antibiotic allergies in pediatric patients could reduce the inappropriate use of second-line antibiotics, improve patient safety and support the broader goal of combating antimicrobial resistance.

### 3.3. Hymenoptera Venom Allergy

#### 3.3.1. Summary of Literature Research

**Figure 3:**
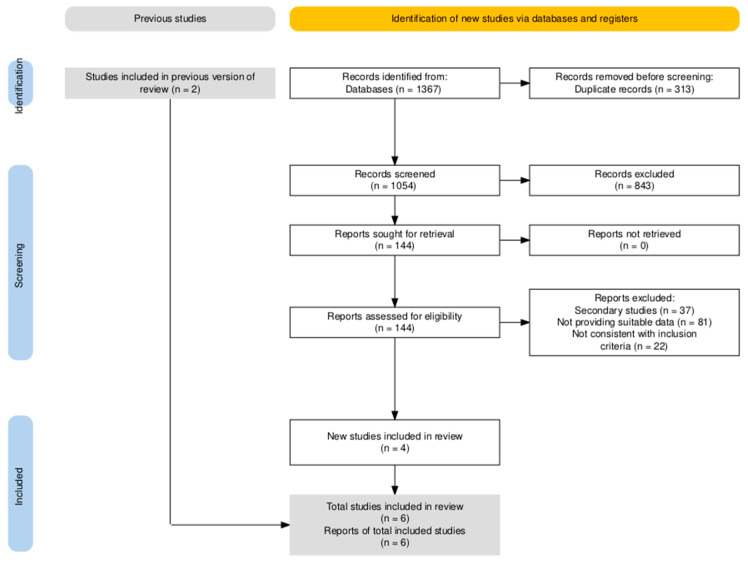
PRISMA flowchart for included studies on hymenoptera venom allergy.

The research was performed over three databases (Embase, Medline, Cochrane) and identified a total of 1367 articles. Detailed study selection process is presented in the PRISMA flow diagram reported in Figure 3.

Briefly, after the removal of duplicated items (n = 313, 22.9%), remaining 1054 articles were screened by title and abstract, with the subsequent identification of 144 suitable entries (10.5% of the original sample). A total of 10 articles were then suggested by expert opinion, but only two of them were eventually consistent with the aims of the literature search. A total of six studies were eventually included into the analyses, all of them being observational studies. Included studies are reported in full details and described in Table A9 and Table A10.

Outcomes considered (depending on the PICO) mainly included:

- Risk of anaphylaxis;
- Diagnostic accuracy to initiate appropriate treatment.

#### 3.3.2. Clinical Questions for Hymenoptera Venom Allergy

##### PICO 1

For children and adolescents with systemic reactions to hymenopteran stings (P), is referral to an allergy specialist (I) recommended over management by the primary care pediatrician (C) to assess the risk of anaphylaxis and initiate preventive treatment (O)?

Evidence (Quantitative analysis): Due to the heterogenous design of retrieved studies, both in terms of targeted population and included outcomes, no quantitative summary of evidence was ultimately performed by means of a meta-analytical approach.

Evidence (Narrative report): Allergic reactions to Hymenoptera stings vary widely in severity and may occasionally be fatal. HVA is a significant cause of morbidity and mortality worldwide, although it is still underestimated from an epidemiological perspective due to the underreporting of many cases [8]. It is estimated that up to 94% of the adult population worldwide are stung at least once in their lifetime. Hymenoptera venom-induced anaphylaxis occurs in 0.3–8.9% of people who are stung. Nevertheless, it remains the leading cause of anaphylaxis, accounting for around 20% of all fatal reactions [8,9,10]. Although children are sensitized less frequently and generally experience milder reactions than adults, likely due to lower venom exposure and fewer comorbidities, the number of pediatric patients who develop systemic reactions remains clinically significant. Epidemiological data are primarily derived from questionnaire-based studies. The prevalence of large local reactions (LLRs) ranges from 0.9% in Italy to 11.5% in Israel, whereas the prevalence of systemic reactions (SRs) has been reported to be below 1% in an Italian study but higher in an Israeli study (6.5%) [58,59]. According to data from the European Anaphylaxis Registry, HVA is the second most common cause of severe anaphylaxis in children (20.2%), following food allergy [60]. Hypersensitivity to hymenopteran venom may result from immunological mechanisms, either IgE-mediated or non-IgE-mediated, or from non-immunological processes. Allergic reactions to Hymenoptera stings are classified into simple local reactions, LLRs, SRs (anaphylactic and non-anaphylactic), toxic reactions and atypical reactions [61]. Several classification systems have been proposed over time to assess the severity of systemic reactions to Hymenoptera stings. The Müller and Ring and Messmer classifications are the most widely adopted of these, although both present certain inherent limitations. The Müller classification does not account for the possible absence of cutaneous symptoms, nor does it consider cases in which cardiovascular shock is the only clinical manifestation. By contrast, the Ring and Messmer classification focuses primarily on cardiovascular collapse, which is considered to be more severe than respiratory involvement [61,62]. A recent literature review [63] classified risk factors for anaphylactic reactions to Hymenoptera stings into two categories: situational and long-term. Several factors in the former category have been confirmed as predictors of fatal outcomes, including delayed administration of epinephrine, being in an upright posture during anaphylaxis, physical exertion during or after being stung, consuming alcohol or acetylsalicylic acid, having concomitant infections, experiencing stress, and being in the menstrual cycle. Within the group of long-term risk factors, there is evidence to suggest an association between systemic mastocytosis and severe anaphylactic reactions. Male sex, cardiovascular comorbidities and older age (over 40 years) also play a significant role due to the higher prevalence of comorbidities and increased risk of systemic reactions compared to children. In the pediatric population, the literature reports conflicting data on the risk factors that predispose individuals to SRs. A recent systematic review highlighted that a previous history of severe SRs and the number of prior stings is significant risk factors for future SRs in children. Increased exposure to a specific allergen, which is often related to lifestyle and environmental factors, has been associated with a higher risk of sensitization and subsequent SRs. Conversely, there is no conclusive evidence of a genetic predisposition in pediatric patients. In this context, ethnicity appears to influence the risk of SRs primarily through lifestyle differences among the studied populations, rather than through true genetic determinants. With respect to sex, currently available data do not indicate a significant difference in SR risk between males and females. Regarding the presence of concomitant allergic conditions, asthma has been identified as the only potential risk factor for Hymenoptera venom–induced SRs [64]. In a cohort of adolescents aged between 13 and 14, subjects suffering from asthma, but also allergic rhinitis and/or atopic dermatitis showed a significantly higher incidence of severe SRs than their non-atopic peers (36.9% vs. 24.8%), suggesting that atopic status represents an additional risk factor [59]. Other studies have shown that only the presence and severity of asthma appear to correlate with the intensity of the systemic response [65,66]. Regarding age, several studies suggest that older children are at increased risk of developing SRs. The mean age at which children experienced anaphylactic reactions was lower among those allergic to honeybee venom. Furthermore, children with a honeybee venom allergy appear to be at a higher risk of SRs. However, the frequency of SRs did not differ significantly between wasp and honeybee allergies. Finally, one study reported that the site of the sting, particularly when it involves the head or neck, may be an additional risk factor for SRs [67]. However, this finding contrasts with observations in the adult population [63]. Depending on the severity of the SR, acute management may include the administration of H1-antihistamines and systemic glucocorticoids. If necessary, self-injectable epinephrine may also be used. These patients therefore require evaluation by allergy specialists who are adequately trained [62]. Numerous guidelines recommend a comprehensive allergological work-up in the presence of a suspected SR. This includes taking a detailed clinical history to identify risk factors for severe reactions, carrying out skin tests [such as SPTs and intradermal tests (IDTs)] and measuring specific serum IgE to identify the causative Hymenoptera species. Additional diagnostic tools may be employed in more complex cases, such as capillary-based allergen-specific immunoassay inhibition (CAP) and the BAT [8,61,62,68,69]. All patients with a history of anaphylaxis, as well as their caregivers, should be given a self-injectable epinephrine device and taught how to recognize and manage the signs and symptoms of anaphylaxis [61,62,68,69]. Children who have been prescribed a self-injectable epinephrine device should carry it with them at all times, including at school. The allergy centre must provide a medical certificate authorizing the administration of the medication during school hours [61]. Following a systemic reaction to a Hymenoptera sting, the patient should be referred to an allergologist for a diagnostic assessment, a prescription for a self-injectable epinephrine device, and an evaluation for venom-specific immunotherapy (VIT). VIT is currently the only treatment capable of altering the natural course of the disease. Studies have shown that patients undergoing VIT report a significantly better quality of life than those managed solely with epinephrine. Previous systemic reactions to hymenopteran venom have a negative impact on quality of life, particularly due to anxiety about potential future stings and fear of fatal outcomes. In children, this emotional burden naturally extends to their caregivers as well [8,61].

**Recommendation** (Table 7): For children and adolescents experiencing systemic reactions to hymenopteran stings, referral to an allergy specialist is recommended rather than being managed by a pediatrician alone. This allows for an accurate diagnosis, identification of risk factors for severe systemic reactions, education of patients on how to manage future stings and the prescription of self-injectable adrenaline. If indicated, it also allows for the initiation of venom-specific immunotherapy. This is the only treatment capable of altering the natural progression of the disease, thereby also improving quality of life.

##### PICO 2

For children and adolescents suspected of having a HVA with systemic reactions (P), is it more strongly recommended that specific diagnostic tests (skin testing and measurement of specific IgE) are performed (I) than clinical follow-up alone (C), in order to improve diagnostic accuracy and guide therapeutic decision-making (O)?

Evidence (Quantitative analysis): Due to the heterogenous design of retrieved studies, both in terms of targeted population and included outcomes, no quantitative summary of evidence was ultimately performed by means of a meta-analytical approach.

Evidence (Narrative report): According to the EAACI guidelines on allergen immunotherapy (AIT) for HVA, AIT is indicated for both children and adults who have experienced systemic allergic reactions extending beyond generalized cutaneous symptoms. Sensitization to the venom of the culprit Hymenoptera species must be documented and confirmed through an appropriate allergological investigation. To initiate AIT, it is essential to accurately identify the venom to which the patient is sensitized and has a clinical reaction to. The diagnostic goals are to determine the reaction type, confirm an IgE-mediated mechanism and identify the causative insect. Correct identification of the offending insect is an essential step in the diagnostic process. Bilò and Giovannini reported that combining an entomological chart with a detailed clinical history during the allergological consultation enabled the culprit insect to be accurately identified in around 73% of cases [8,70]. In a multicentre study, Baker et al. demonstrated that allergologists have significantly greater expertise in identifying insects of the order Hymenoptera than their non-allergologist colleagues [71]. Skin testing using venom extracts and measurement of sIgE, should be performed in all patients with a history of systemic reactions, as recommended by all major guidelines [8,61,62,68,69,72,73]. For patients with significant local reactions, diagnostic testing is optional and should consider the patient’s age (60% of systemic reactions in children are mild) as well as individual risk factors. These include a high likelihood of future stings; occupational exposure (e.g., beekeeping); pre-existing cardiovascular or respiratory diseases; use of beta-blockers or Angiotensin-Converting Enzyme (ACE) inhibitors; the type of insect (in the Mediterranean area, the risk of potentially fatal reactions is approximately three times higher for hornet stings than for other wasps and honeybees); the location of the sting; elevated baseline serum tryptase levels; and the presence of mastocytosis [8]. European guidelines [72,73] recommend a stepwise approach to skin testing. This starts with the SPT, followed by the IDT if the SPT is negative. An IDT should also be performed if the SPT is positive in order to determine the intradermal endpoint, which is useful for follow-up in VIT. In fact, the SPT is less sensitive than the IDT. A recent study of 301 patients allergic to Vespula venom showed that the SPT alone identified 49% of cases, whereas the combination of the SPT and IDT allowed diagnosis in 94% of cases. This highlights the importance of performing both tests [74]. Skin testing should be conducted at least two weeks after the sting to minimize the risk of false-negative results due to the refractory period. It should also be repeated after one to two months if the initial results are negative despite a convincing clinical history of a systemic reaction [8]. The severity of the reaction is not correlated with skin test reactivity. Some authors [62] have suggested that skin testing may be omitted in the following circumstances: (1) when skin testing poses a significant risk; (2) when performing the test would significantly impact the patient; and (3) when a conclusive result has already been obtained through in vitro testing. However, skin tests should be performed when sIgE (specific immunoglobulin E) testing is negative, or when there is a discrepancy between the clinical history and laboratory findings. The sensitivity of serum testing with whole venom extracts is generally lower than that of skin tests. Up to 20% of patients with positive skin test results may have negative in vitro sIgE results, whereas approximately 10% of patients with negative skin test results may have positive in vitro sIgE results [61,70]. Therefore, current guidelines recommend carrying out both types of tests. Although specific IgE antibodies may be detectable shortly after the sting, the optimal timing for measurement is between one- and four-weeks post-sting. This may sometimes allow identification of the culprit insect. In cases of double positivity for sIgE but a positive IDT result for only one venom, VIT should be performed using the venom that tested positive in the IDT. This is because the IDT is not influenced by cross-reactive carbohydrate determinants (CCDs) [5]. One of the main diagnostic challenges arises when patients who have been stung by an unidentified insect show positive results for two different allergens in testing. Dual sensitization to Apis mellifera and Vespula occurs in 25–40% of cases. The availability of recombinant allergens enables molecular diagnosis and differentiation between true double sensitization and cross-reactivity. This approach helps to select the most appropriate venom for VIT and avoids the need for dual-venom treatment. In unclear cases, a BAT should also be performed. The BAT is particularly recommended for patients with a convincing clinical history but negative skin and sIgE tests, as well as in cases with inconclusive molecular allergen results and double sensitization [75]. In a recent observational study, the BAT demonstrated high sensitivity and a positive predictive value. The clinical sensitivities were 95.5% for Apis mellifera, 95.7% for sIgE and 48.4% for SPT; for Vespula vulgaris, these values were 83.3%, 100% and 33.3%, respectively. Based on these results, systemic reaction prediction was ranked as follows: sIgE > BAT > SPT. Dual sensitization was observed in 39.5% of patients using the diagnostic provocation test (DPT) and in 22% using sIgE testing; however, none showed dual sensitization with the BAT [75]. Therefore, the BAT plays a key role in guiding therapeutic decisions for patients with dual sensitization. Another method of distinguishing true dual sensitization from cross-reactivity is CAP inhibition; however, this test is expensive and can be difficult to interpret. In a recent observational study, Tischler et al. demonstrated that the presence of dominant sIgE with a ratio of 5:1 or higher in allergic patients with dual sensitization represents a reliable indicator for identifying the culprit venom. Based on these findings, the authors suggest that additional diagnostic investigations, such as the BAT and CAP inhibition tests, should be reserved for cases in which dual sensitization to whole venoms presents a ratio below 5:1 [76]. In cases of severe systemic reactions with confirmed dual positivity, VIT involving both venoms is recommended. Due to the risk of severe systemic reactions and their low negative predictive value, live hymenoptera challenges should not be performed [62,73]. Baseline serum tryptase levels should be measured in all patients who experience a systemic reaction to identify those at increased risk of severe reactions due to clonal mast cell disorders [69,72]. AIT is currently the only treatment that can modify the natural progression of the disease. If left untreated, the disease poses a life-threatening risk.

Recommendation (Table 8): Due to the moderate quality of evidence, a consensus-based recommendation rather than an evidence based was framed. For children and adolescents suspected of having a HVA and systemic reactions, specific diagnostic tests are preferred over simple clinical follow-up, as they improve diagnostic accuracy and inform therapeutic decisions.

## 4. Discussion

This consensus document provides an updated, evidence-based framework for improving the appropriateness of diagnostic prescriptions in three key domains of pediatric allergology, anaphylaxis, drug allergy, and HVA. Across all areas, the findings underscore the need to reduce unwarranted diagnostic variation, streamline clinical pathways, and promote rational use of healthcare resources. The multidisciplinary composition of the expert panel, involving clinicians from primary care, emergency medicine, allergology, dermatology, immunology, psychology, and nursing, ensures that the recommendations reflect real-world practice needs and are aligned with the priorities of families, clinicians, and the healthcare system.

For children with suspected anaphylaxis, the evidence consistently supports the value of structured diagnostic evaluation over clinical follow-up alone. Measurement of acute and baseline serum tryptase can confirm mast-cell activation and assist in distinguishing true anaphylaxis from mimicking conditions, particularly in cases with an uncertain presentation. Although elevated tryptase is observed only in a subset of pediatric reactions, its incorporation into deferred-urgency evaluations enhances diagnostic reliability and helps identify individuals at increased risk due to underlying mast-cell disorders [2,21].

SPT using extracts and fresh foods remains a first-step tool to identify sensitization relevant to food-induced anaphylaxis. Results must, however, be interpreted alongside clinical history, as sensitization does not always reflect clinically reactive disease [2,38]. In selected cases, CRD provide additional molecular precision, supporting risk stratification and identification of specific allergenic proteins associated with more severe reactions. While CRD is not a stand-alone test and cannot precisely predict reaction severity, its use within specialist settings can meaningfully refine diagnostic and preventive strategies [42]. Ultimately, specialist referral after anaphylaxis remains essential to ensure allergen identification, prescription and training in AAI use, and formulation of individualized action plans [2].

Across drug allergy, particularly in suspected β-lactam allergy, the consensus reinforces a fundamental clinical message: reliance on patient history alone results in substantial overdiagnosis and unnecessary restriction of first-line antibiotics. Such over-avoidance contributes to increased use of second-line agents, higher treatment costs, and the proliferation of antimicrobial resistance. Integrating skin testing, specific IgE assays, and, most importantly, OPT significantly improves diagnostic accuracy. Evidence indicates that most children labelled as allergic can safely tolerate β-lactams when evaluated using structured protocols [4,47,48].

Direct OPT in low-risk pediatric patients has demonstrated both safety and strong diagnostic yield, and when negative, allows for immediate de-labelling. Although OPT can generate apprehension among parents, its clinical benefits, long-term safety implications, and positive impact on antimicrobial stewardship are substantial. Implementing these pathways requires access to dedicated allergy services, but the long-term cost savings, through correct antibiotic use and prevention of unnecessary referrals, support their widespread adoption [4,47,48].

For children with systemic reactions to hymenopteran stings, the consensus highlights the critical role of referral to an allergy specialist. Accurate diagnosis relies on a detailed clinical history supported by SPT or intradermal testing and determination of venom-specific IgE. In selected cases with dual sensitization or uncertain results, BAT or inhibition assays may provide additional clarity, although access may be limited.

Specialist evaluation enables appropriate prescription of AAI devices, reinforces education on sting avoidance and emergency management, and, crucially, provides access to VIT. As the only intervention capable of altering the natural course of venom allergy, VIT substantially reduces the risk of future systemic reactions and has been shown to markedly improve quality of life for both patients and caregivers. Despite these benefits, disparities in access to pediatric allergy centres remain a barrier and require coordinated efforts at the health-system level [62,68,69].

Taken together, these recommendations offer a coherent and practical roadmap for standardizing diagnostic pathways in pediatric allergology. By emphasizing targeted testing, multidisciplinary evaluation, and specialist involvement when indicated, the consensus promotes a more equitable allocation of healthcare resources and helps mitigate inappropriate practice patterns. Importantly, the recommendations also address parental expectations and the high value placed on diagnostic clarity, safety, and prevention of recurrence. Broader implementation will require dissemination of training materials, strengthening of referral networks, and investment in pediatric allergy services, but the anticipated clinical and economic benefits justify these efforts.

Despite its strengths, this work has several limitations. First, although the recommendations are grounded in systematic reviews, the evidence base for some clinical questions, particularly within pediatric drug allergy and HVA, remains limited by the scarcity of randomized trials and the predominance of observational studies. This may introduce bias and restrict the certainty of some estimates. Second, heterogeneity in study design, diagnostic protocols, and outcome definitions across the included literature limited the feasibility of performing meta-analyses for included PICOs. Moreover, the limited quality of evidence resulting from the aforementioned limits forced the Authors to frame consensus-based rather than evidence-based recommendations. Third, the availability of diagnostic tools such as CRD or BAT varies considerably across centres, which may affect the generalizability and implementation of certain recommendations. Fourth, expert consensus, while valuable, may reflect context-specific practice patterns within the Italian healthcare system and may not fully represent international diversity in clinical environments. Finally, although the multidisciplinary panel ensures comprehensive perspectives, the lack of direct patient-reported outcome data within the systematic review limits the ability to quantify the psychosocial impact of different diagnostic strategies.

## 5. Conclusions

This consensus document provides a comprehensive, evidence-based framework to support more appropriate, consistent, and clinically effective diagnostic practices in pediatric anaphylaxis, drug allergy, and hymenoptera venom allergy. By integrating current scientific evidence with expert multidisciplinary insight, these recommendations aim to reduce unwarranted variability, improve diagnostic precision, and ensure timely access to specialist evaluation when needed.

## Figures and Tables

**Table 1:** Summary of evidence and main recommendations on the role of deferred-urgency allergy evaluation for identifying the triggering factor, prescribe an adrenaline auto-injector (AAI) with appropriate training in its use, and provide strategies for preventing and managing recurrences, compared to the routine follow-up with a primary care pediatrician.

Quality of Evidence (GRADE)	Strength of Recommendation	Balance of Benefits and Risks	Values and Preferences	Resource Use Implementation Impact
Moderate	Strong recommendation for referral to a specialist in deferred urgency	Benefits: identification of the trigger, AAI prescription and training, and provision of an action plan. Risks: cost and access time. The benefit–risk balance is clearly favourable.	Families place a high value on preventing recurrence and safety; overall acceptability is high.	It requires an established allergy referral network and access to training materials; it may reduce emergency department visits and recurrence-related costs.

**Table 2:** Summary of evidence and main recommendations on the role of measurement of serum tryptase over clinical follow-up alone, to facilitate diagnosis, stratify risk (e.g., identify underlying conditions such as systemic mastocytosis or predisposition to more severe reactions) and improve long-term management (i.e., preventing new episodes).

Quality of Evidence (GRADE)	Strength of Recommendation	Balance of Benefits and Risks	Values and Preferences	Resource Use Implementation Impact
Moderate	Strong recommendation to measure serum tryptase (acute + baseline)	Benefits: supports objective diagnosis, allows risk stratification, and may reveal underlying mastocytosis or Mast Cell Activation Syndrome (MCAS). Risks: low cost and need for precise timing.	High value for diagnostic confirmation and management planning; good acceptability (blood test).	Available in most laboratories; requires logistical coordination for time-sensitive sampling; modest economic impact.

**Table 3:** Summary of evidence and main recommendations on the role of skin prick testing (SPT) with both commercial extracts and fresh foods recommended over clinical follow-up alone (C) to prevent new episodes, improve diagnostic accuracy, avoid unnecessary elimination diets, and enhance quality of life among children and adolescents with suspected food allergy–related anaphylaxis.

Quality of Evidence (GRADE)	Strength of Recommendation	Balance of Benefits and Risks	Values and Preferences	Resource Use Implementation Impact
Moderate	Weak/conditional recommendation in favour of SPT (extracts + fresh food) when indicated	Benefits: improved diagnostic accuracy and avoidance of unnecessary diets. Risks: false positives, requires expert interpretation.	Families seek to clarify food-related responsibilities and reduce dietary restrictions; high value on quality of life (QoL).	Low-cost and widely available tests; expertise is required for the selection of extracts/fresh food and interpretation.

**Table 4:** Summary of evidence and main recommendations on the role of measurement of specific IgE by means of component-resolved diagnostics (CRD), rather than relying solely on clinical follow-up, in order to identify individual risk profiles, personalize prevention strategies, and prevent new episodes among children and adolescents suspected of having anaphylaxis.

Quality of Evidence (GRADE)	Strength of Recommendation	Balance of Benefits and Risks	Values and Preferences	Resource Use Implementation Impact
Low to Moderate	Weak/conditional recommendation in favour of specific IgE measurement using CRD	Benefits: risk profiling (e.g., storage proteins/profilins/lipid transfer proteins), personalized prevention and AAI use. Risks: cost, potential for over-interpretation.	Clinicians and families value risk stratification and personalized advice; good acceptability.	Variable availability; higher cost than standard tests; implementation in specialized centres with experience.

**Table 5:** Summary of evidence and main recommendations on the role of skin tests, oral provocation tests (OPT) and specific serological assays for improving diagnostic accuracy, reducing misdiagnoses and decreasing the use of less effective alternative treatments, compared with a diagnosis based solely on clinical history, among children and adolescents suspected of having a beta-lactam antibiotic allergy.

Quality of Evidence (GRADE)	Strength	Balance of Benefits/Risks	Values and Preferences	Impact on Resources/Implementation
Moderate	Strongly recommended use of diagnostic tests over medical history alone	Significantly greater benefits (improved diagnostic accuracy, fewer misdiagnoses, reduced use of alternative antibiotics)	Parents and clinicians supportive, although OPT may cause apprehension	Requires centres equipped for tests/OPT; higher initial cost but cost-effective in the long term

**Table 6:** Summary of evidence and main recommendations on the role of a targeted diagnostic pathway before prescribing alternative antibiotics for reducing the inappropriate use of second-line agents, compared with empirically prescribing unrelated antibiotics among children and adolescents suspected of having an antibiotic allergy.

Quality of Evidence (GRADE)	Strength	Balance of Benefits/Risks	Values and Preferences	Impact on Resources/Implementation
N.A.	N.A.	Benefits possibly outweigh risks (improved diagnostic accuracy, fewer incorrect diagnoses, reduced use of alternative antibiotics)	Parents and clinicians supportive	Requires centres equipped with specific consultations; possible delay in application of therapy but probably sustainable

**Table 7:** Summary of evidence and main recommendations on the role of the referral to an allergy specialist over management by the primary care pediatrician in order to assess the risk of anaphylaxis and initiate preventive treatment among children and adolescents with systemic reactions to hymenopteran stings.

Quality of Evidence (GRADE)	Strength of Recommendation	Balance of Benefits and Risks	Values and Preferences	Resource Use/Implementation Impact
**Moderate-high**	Strong in favour of specialist evaluation	Although greater use of specialist resources compared with management by a pediatrician alone, it is highly cost-effective for the prevention of acute events and hospitalizations.	Families and clinicians place great importance on anaphylaxis prevention and safety. High acceptability among families and pediatricians, strong endorsement from clinicians and the healthcare system.	Integration into regional care pathways and improvement in the availability of pediatric allergy centres are required.

**Table 8:** Summary of evidence and main recommendations on the role of specific diagnostic tests (skin testing and measurement of specific IgE) over clinical follow-up alone, in order to improve diagnostic accuracy and guide therapeutic decision-making, among children and adolescents suspected of having a hymenoptera venom allergy with systemic reactions.

Quality of Evidence (GRADE)	Strength of Recommendation	Balance of Benefits and Risks	Values and Preferences	Resource Use Implementation Impact
Moderate	Strong recommendation in favour of specific diagnostic tests	Balance in favour of the use of specific diagnostic tests Minimal risks: local reactions to skin tests; anxiety related to the investigation; risk of false positives/negatives	Families and doctors tend to prefer tests that provide diagnostic clarity and certainty in treatment decisions Generally high acceptability by families and clinicians	Easily implementable in allergy centres; requires shared pathways and pediatric training. Additional costs for diagnostic tests but largely justified by improved treatment targeting and reduction in adverse events. Possible disparities in access to specialized laboratories and advanced tests (e.g., BAT—basophil activation test).

## Data Availability

The original contributions presented in this study are included in the article/Supplementary Material. Further inquiries can be directed to the corresponding author(s).

## Supplementary Materials

The following supporting information can be downloaded at: https://www.mdpi.com/article/10.3390/jcm15020678/s1, Table S1. PRISMA checklist.

## Appendix A

**Table A1:** Summary of search strategies.

Topic	PICO	PubMed	Embase
Anaphylaxis	1	(“Ambulatory Care”[Mesh] OR “Day Care, Medical”[Mesh] OR “home visit*” OR “outpatient*”) AND (“Anaphylaxis”[Mesh] OR “Hypersensitivity, Immediate”[Mesh]) AND (“Emergency Medical Services”[Mesh] OR “Self Administration”[Mesh] OR “Epinephrine”[Mesh]) AND (“Tryptases”[MeSH] OR tryptase[tiab] OR “serum tryptase”[tiab] OR “mast cell tryptase”[tiab] OR “tryptase level”[tiab] OR “baseline tryptase”[tiab] OR “acute tryptase”[tiab]) AND (“Child”[MeSH] OR “Adolescent”[MeSH] OR child*[tiab] OR pediatric*[tiab] OR pediatric*[tiab] OR adolescen*[tiab])	(‘ambulatory care’/exp OR ‘ambulatory care’ OR ‘day care’ OR ‘home visit’ OR ‘outpatient’) AND (‘anaphylaxis’ OR ‘hypersensitivity’) AND (‘emergency health service’ OR ‘drug self administration’ OR ‘epinephrine’) AND (‘tryptase’) AND ([adolescent]/lim OR [child]/lim OR [newborn]/lim OR [preschool]/lim)
	2	(“Anaphylaxis”[MeSH] OR anaphylaxis[tiab] OR anaphylactic[tiab]) AND (“Tryptases”[MeSH] OR tryptase[tiab] OR “serum tryptase”[tiab] OR “mast cell tryptase”[tiab] OR “tryptase level”[tiab] OR “baseline tryptase”[tiab] OR “acute tryptase”[tiab]) AND (“Child”[MeSH] OR “Adolescent”[MeSH] OR child*[tiab] OR pediatric*[tiab] OR pediatric*[tiab] OR adolescen*[tiab])	(‘anaphylaxis’ OR ‘hypersensitivity’) AND (‘tryptase’ OR ‘tryptase test kit’ OR ‘tryptase blood level’) AND ([adolescent]/lim OR [child]/lim OR [newborn]/lim OR [preschool]/lim)
	3	((“Food Hypersensitivity”[MeSH] OR “Food Hypersensitivity”[tiab] OR “food allergy”[tiab] OR “food-induced allergy”[tiab] OR “food-induced anaphylaxis”[tiab] OR “food allergy–related anaphylaxis”[tiab]) AND (“Anaphylaxis”[MeSH] OR anaphylaxis[tiab]) AND (“Skin Tests”[MeSH] OR “skin prick test”[tiab] OR “skin-prick test”[tiab] OR “SPT”[tiab] OR “prick test”[tiab]) AND (“Child”[MeSH] OR “Adolescent”[MeSH] OR child*[tiab] OR pediatric*[tiab] OR pediatric*[tiab]))	(‘food allergy’ AND (‘anaphylaxis’ OR ‘hypersensitivity’)) AND ([adolescent]/lim OR [child]/lim OR [newborn]/lim OR [preschool]/lim) AND (‘skin test’ OR ‘prick test’)
	4	(“Anaphylaxis”[MeSH] OR anaphylaxis[tiab] OR anaphylactic[tiab]) AND (“Immunoglobulin E”[MeSH] OR “specific IgE”[tiab] OR “specific IgE”[MeSH] OR “component resolved”[tiab] OR “component-resolved”[tiab] OR “component resolved diagnostics”[tiab] OR “component-resolved diagnostics”[tiab] OR CRD[tiab] OR “molecular allergology”[tiab]) AND (“Child”[MeSH] OR “Adolescent”[MeSH] OR child*[tiab] OR pediatric*[tiab] OR pediatric*[tiab] OR adolescen*[tiab])	(‘anaphylaxis’ OR ‘hypersensitivity’) AND ([adolescent]/lim OR [child]/lim OR [newborn]/lim OR [preschool]/lim) AND (‘immunoglobulin’ OR ‘immunoglobulin e’ OR ‘component resolved diagnosis’ OR ‘component resolved diagnostics’ OR (molecular AND allergology)
Drug Allergy	1	(“Hypersensitivity/drug therapy”[MeSH] OR “Drug Hypersensitivity”[MeSH] OR “beta-lactams” [tiab] OR “beta lactam”[tiab] OR “penicillin allergy”[tiab] OR “penicillin hypersensitivity”[tiab] OR “cephalosporin allergy”[tiab] OR “drug allergy”[tiab]) AND (“Skin Tests”[MeSH] OR “skin prick”[tiab] OR “prick test”[tiab] OR intradermal[tiab] OR “intradermal test”[tiab]) AND (“Drug Provocation Test”[tiab] OR “oral provocation test”[tiab] OR “oral challenge”[tiab] OR “drug challenge”[tiab]) AND (“Immunoglobulin E”[MeSH] OR “specific IgE”[tiab] OR sIgE[tiab] OR “drug-specific IgE”[tiab]) AND (diagnos*[tiab] OR “diagnostic accuracy”[tiab] OR “misdiagnosis”[tiab] OR “incorrect diagnosis”[tiab] OR “false allergy”[tiab] OR sensitivity[tiab] OR specificity[tiab] OR “alternative treatment”[tiab] OR “treatment alternatives”[tiab] OR “antibiotic stewardship”[tiab]) (“Anaphylaxis”[MeSH] OR anaphylaxis[tiab] OR anaphylactic[tiab]) AND (“Tryptases”[MeSH] OR tryptase[tiab] OR “serum tryptase”[tiab] OR “mast cell tryptase”[tiab] OR “tryptase level”[tiab] OR “baseline tryptase”[tiab] OR “acute tryptase”[tiab]) AND (“Child”[MeSH] OR “Adolescent”[MeSH] OR child*[tiab] OR pediatric*[tiab] OR pediatric*[tiab] OR adolescen*[tiab])	“(‘drug hypersensitivity’ OR ‘penicillin allergy’ OR ‘beta lactam allergy’) AND ((‘prick test’ OR ‘immunoglobulin e’ OR ‘immunological procedures’ OR ‘anamnesis’) AND (‘follow up’ OR ‘accuracy’ OR ‘diagnostic accuracy’ OR ‘therapy’))”
	2	((“beta-Lactams”[Mesh] OR “beta Lactam Antibiotics”[Mesh] OR “beta Lactam Antibiotics” [Pharmacological Action]) OR “Penicillins”[Mesh]) AND (“Drug Hypersensitivity Syndrome”[Mesh] OR “Drug Hypersensitivity”[Mesh]) AND (“Skin Tests”[Mesh] OR “Immunoglobulin E”[Mesh] OR “Follow-Up Studies”[Mesh] OR “Medical History Taking”[Mesh]) AND (“Case Management”[Mesh] OR “Patient Care Management”[Mesh] OR “Missed Diagnosis”[Mesh] OR “Quality of Life”[Mesh]) (“Anaphylaxis”[MeSH] OR anaphylaxis[tiab] OR anaphylactic[tiab]) AND (“Tryptases”[MeSH] OR tryptase[tiab] OR “serum tryptase”[tiab] OR “mast cell tryptase”[tiab] OR “tryptase level”[tiab] OR “baseline tryptase”[tiab] OR “acute tryptase”[tiab]) AND (“Child”[MeSH] OR “Adolescent”[MeSH] OR child*[tiab] OR pediatric*[tiab] OR pediatric*[tiab] OR adolescen*[tiab])	(‘drug hypersensitivity’ OR ‘penicillin allergy’ OR ‘beta lactam allergy’) AND ((‘prick test’ OR ‘immunoglobulin e’ OR ‘immunological procedures’ OR ‘anamnesis’) AND (‘follow up’ OR ‘accuracy’ OR ‘diagnostic accuracy’ OR ‘therapy’))”
Hymenoptera Venom Allergy	1	(“Arthropod Venoms”[Mesh] OR “Bee Venoms”[Mesh] OR “Venom Hypersensitivity”[Mesh]) AND ((“Allergy and Immunology”[Mesh] AND (“Ambulatory Care”[Mesh] OR “Office Visits”[Mesh])) OR (“Pediatrics”[Mesh] AND (“Ambulatory Care”[Mesh] OR “Office Visits”[Mesh])) AND (“Anaphylaxis”[Mesh]) OR “Recurrence”[Mesh] OR “Immunomodulation”[Mesh] OR “Immunotherapy, Active”[Mesh]) (“Anaphylaxis”[MeSH] OR anaphylaxis[tiab] OR anaphylactic[tiab]) AND (“Tryptases”[MeSH] OR tryptase[tiab] OR “serum tryptase”[tiab] OR “mast cell tryptase”[tiab] OR “tryptase level”[tiab] OR “baseline tryptase”[tiab] OR “acute tryptase”[tiab]) AND (“Child”[MeSH] OR “Adolescent”[MeSH] OR child*[tiab] OR pediatric*[tiab] OR pediatric*[tiab] OR adolescen*[tiab])	((‘hymenoptera’/exp OR ‘hymenoptera’) OR ‘insect venom’ OR ‘hymenoptera venom’ OR ‘hymenoptera venom allergy’) AND (‘immunology’ AND (‘outpatient’ OR ‘ambulatory care’ OR ‘hospital visit’) OR (‘pediatrics’ AND (‘outpatient’ OR ‘ambulatory care’ OR ‘hospital visit’))) AND (‘immunomodulation’ OR ‘immunotherapy’ OR ‘anaphylaxis’ OR ‘relapse’ OR ‘recurrent disease’ OR ‘recurrence risk’)
	2	(“Hymenoptera Venoms”[MeSH] OR “Insect Stings”[MeSH] OR hymenoptera[tiab] OR “insect sting”[tiab]) AND (“Skin Tests”[MeSH] OR prick[tiab] OR intradermal[tiab]) AND (“Immunoglobulin E”[MeSH] OR “specific IgE”[tiab] OR sIgE[tiab]) AND (“Basophil Activation Test”[tiab] OR BAT[tiab]) AND (diagnos*[tiab] OR “diagnostic accuracy”[tiab] OR sensitivity[tiab] OR specificity[tiab]) (“Anaphylaxis”[MeSH] OR anaphylaxis[tiab] OR anaphylactic[tiab]) AND (“Tryptases”[MeSH] OR tryptase[tiab] OR “serum tryptase”[tiab] OR “mast cell tryptase”[tiab] OR “tryptase level”[tiab] OR “baseline tryptase”[tiab] OR “acute tryptase”[tiab]) AND (“Child”[MeSH] OR “Adolescent”[MeSH] OR child*[tiab] OR pediatric*[tiab] OR pediatric*[tiab] OR adolescen*[tiab])	(‘hymenoptera venom allergy’ OR ‘bee venom extract’ OR ‘insect allergy’) AND (‘prick test’ OR ‘immunoglobulin e’ OR ‘immunological procedures’ OR ‘anamnesis’) AND (‘follow up’ OR ‘accuracy’ OR ‘diagnostic accuracy’ OR ‘therapy’)

**Table A2:** Appraisal of the risk of Bias of observational studies according to Newcastle Ottawa Scale (range 0–9) [14,77].

Author	Year	D1	D2	D3	D4	D5	D6	D7	D8	D9	Score
Anaphylaxis											
De Schryver S et al. [33]	2016	+		+	+	+	+	+	+		7
Cavkaytar et al. [34]	2016	+	+	+	+	+		+			6
Le M. et al. [78]	2018	+		+	+			+	+		5
Vetander M. et al. [38]	2016	+		+	+			+	+		5
Clark E. et al. [32]	2023	+		+	+	+		+			5
Burrell S. et al. [27]	2021	+		+	+			+	+	+	6
Drug allergy											
Moral et al. [53]	2022	+		+	+		+	+	+	6	1
Goh et al. [49]	2021	+		+	+		+	+	+	6	1
Labrosse et al. [52]	2020	+		+	+		+	+	+	6	1
Mill et al. [57]	2016	+		+	+		+	+	+	6	1
Hymenoptera Venom Allergy											
Worm et al. [60]	2014	+			+	+					3
Quercia et al. [58]	2014	+	+			+	+				4
Graif et al. [79]	2009	+	+		+	+	+				5
Tischler et al. [76]	2025	+	+	+	+		+	+			6
Baker et al. [71]	2016			+	+	+	+				4
Kokcu Karadag et al. [75]	2024	+			+						2
Vos et al. [74]	2013	+	+	+	+		+	+			6

**Table A3:** Appraisal of the risk of bias of randomized controlled trials included into the analyses according to the National Toxicology Program (NTP)’s Office of Health Assessment and Translation (OHAT) handbook and respective risk of bias (ROB) tool. Note: D1: possibility of selection bias; D2: exposure assessment; D3: outcome assessment; D4: confounding factors; D5: reporting bias; D6: other bias; : definitively high; : probably high; ☺: probably low; ☺☺: definitively low.

Study	D1	D2	D3	D4	D5	D6
Umasunthar T. et al. 2015 [25]	☺☺	☺☺	☺		☺	
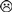
Kukkonen et al. 2016 [44]	☺☺	☺☺	☺		☺	
Van Veen et al. 2016 [45]	☺☺	☺☺	☺	☺	☺	

**Table A4:** Original studies included in the present Good Clinical Practice Report on anaphylaxis (Note: CS = cross-sectional study; CC = case–control study; P = prospective design; R = retrospective design, S = single centre, M = multicenter).

Authors	Year	Type of Study	Objectives	Population (General Characteristics)	Sample Size	Outcome	Results (Quantitative Elements)	Results (Description)	Limitations	Pico
Clark E. et al. [32]	2023	CS, R, M	To assess the appropriateness of using epinephrine in children experiencing anaphylaxis.	Children (aged 0–18) who presented with suspected anaphylaxis at the Pediatric Emergency Department of Montpellier University Hospital between 2016 and 2020.	1.056 Children	To assess whether patients with anaphylaxis received adrenaline as first-line treatment, verify the appropriateness of prescribing adrenaline auto-injectors upon discharge.	Of the 224 children diagnosed with anaphylaxis, only 17.0% received an adrenaline/epinephrine injection, while 57.1% consulted an allergist after the acute episode. Upon discharge from the emergency department, 63 patients (28.1%) were prescribed an auto-injector.	There was inadequate clinical management (less than half of the children received adrenaline) and insufficient preparation of patients and families for preventing future anaphylactic reactions (adrenaline auto-injectors were not prescribed as widely upon discharge).	The study is based on previously recorded clinical data; single-centre study.	1
Burrell S. et al. [27]	2021	CS, R, M	To assess the impact of self-administering adrenaline during oral challenge tests on the health-related quality of life (HRQoL) and self-efficacy of children with a peanut allergy.	Children (aged 8–16) who experienced reactions during oral provocation tests at a hospital’s specialist allergy centre.	56 Children	Health-related quality of life (HRQoL); self-efficacy in managing anaphylaxis; emotional responses to experiencing anaphylaxis in a controlled hospital setting; the impact of self-administering adrenaline on reducing fear and anxiety; the rate of successful self-injections in real-life clinical circumstances.	There was an average improvement of 2.6 points in HRQoL scores and an average increase of 4.1 points in self-efficacy scores after the food challenge. There was also an average improvement of 10.3 points in the child’s HRQoL based on parental opinion. The occurrence of anaphylaxis during the food challenge did not negatively impact HRQoL or self-efficacy.	Self-administration of adrenaline improves HRQoL and self-efficacy. Parents perceive an even greater impact. Encouragement for autonomous use is provided during hospital challenges.	Small sample size; secondary analysis; selection bias; short follow-up period; subjective outcome measures; and limited data on adrenaline usage experience.	1
Le M. et al. [77]	2019	CS, P, M	To evaluate and compare the demographic and clinical characteristics, as well as the management, of cases of anaphylaxis without identifiable triggers in adults and children in Canada.	Adults and children with anaphylaxis (mean age 9.0 years, interquartile range 5.0–14.3, for children and 38.1 years, interquartile range 28.1–51.3 for adults) without identifiable triggers recorded between 2011 and 2018 in the emergency departments of eight centres in Canada as part of the Cross Canada Anaphylaxis Registry (C CARE).	222 children (aged 0–18) and 73 adults	The clinical management and long-term follow-up of patients who have experienced anaphylaxis for which no trigger was identified.	A total of 136 patients received adrenaline in the pre-hospital setting, with a higher percentage of children than adults being treated with adrenaline (74 and 10 cases, respectively). In the emergency department, antihistamines and corticosteroids were used more often in adults than in children. Tryptase levels were measured in only 26 cases (9.8%), and only two patients underwent periodic tryptase monitoring, one of whom had an elevated tryptase level at the time of the reaction. Adrenaline auto-injectors were prescribed in 185 cases, and 40 patients had already received a prescription. Fifty-three patients (43 children and 10 adults) were seen by an allergist or an internist/otorhinolaryngologist specializing in allergology.	There were low referral rates to allergists; children were referred more often than adults. Treatment was inconsistent and frequently suboptimal (there was excessive use of antihistamines and reduced use of adrenaline). Treatment was better for children than adults. Tryptase levels were not measured in most patients at the time of the reaction.	The results are limited to eight centres. Participant recruitment was conducted primarily in academic institutions, where participants are more likely to consult allergists or specialists practising allergy medicine. Recruitment was conducted in the province of Quebec, as three sites in Quebec participated in this study.	1
De Schryver, S et al. [33]	2016	CS, P, S	To assess tryptase levels in children presenting with anaphylaxis, to identify factors associated with elevated tryptase levels, and to evaluate the difference between tryptase levels during the acute reaction and levels post-reaction baseline.	Children (aged 0–18) who presented to the emergency department of the Montreal Children’s Hospital with a diagnosis of anaphylaxis between April 2011 and April 2015	203 Children	The usefulness of tryptase levels as a diagnostic biomarker in pediatric anaphylaxis.	During the reaction, 19.2% of children had tryptase levels of at least 11.4 μg/L. Levels above the threshold (2 ng/mL + 1.2 x baseline or post-reaction tryptase level) were found in 85.7% of severe reactions, 54.2% of moderate reactions and 69.2% of mild reactions. Of the 68 children for whom post-reaction tryptase levels were measured, the mean level at the time of the reaction was 9.9 μg/L, compared to 3.6 μg/L post-reaction, representing a difference of 6.3 μg/L.	Severe reactions were more frequently associated with elevated tryptase levels. However, many children with clinically evident anaphylaxis had normal tryptase levels, indicating that this biomarker has limited sensitivity. Tryptase levels are generally higher during the reaction than the post-reaction baseline, confirming that increased tryptase is a marker of mast cell activation during anaphylaxis.	Only 68 patients had measurements taken during and after the reaction: single-centre study	2
Cavkaytar et al. [34]	2016	CS, R, M	To determine whether baseline serum tryptase levels pose a risk of immediate-type drug hypersensitivity reactions in children.	Children (aged 0–18 years) admitted to the Pediatric Allergy Department at Hacettepe University between January 2012 and August 2015, who had a history of suspected immediate drug hypersensitivity with an onset within six hours of taking the responsible drug.	345 Children	The usefulness of baseline serum tryptase levels as a marker of risk for immediate drug hypersensitivity in children.	A total of 106 children (30.7%) had a history of drug hypersensitivity reaction (DHR), as confirmed by skin and/or provocation tests. The median baseline tryptase level (interquartile range) of patients with DHR was 2.6 μg/L, with a maximum level of 8.2 μg/L. There was no significant difference in baseline tryptase levels between patients who were hypersensitive to the drug, with or without anaphylaxis, [2.6 (2.0–3.6) μg/L vs. 2.8 (1.6–4.3) μg/L, *p* > 0.05].	Basal tryptase levels are not an effective predictor of immediate allergic reactions to drugs in children. Immediate drug hypersensitivity can occur even when these levels are normal.	The absence of serial measurements of serum tryptase levels in patients who are hypersensitive to the drug during actual hypersensitivity reactions to the drug; single-centre study.	2
Vetander M. et al. [38]	2016	CS, P, M	To assess the incidence of food anaphylaxis among adolescents and identify the associated risk factors.	Children aged 0–16 were recruited at birth (1994–1996) in four districts of Stockholm and were then followed up until they reached the age of 16.	3153 Children	Incidence of food anaphylaxis among adolescents, and identification of associated risk factors.	The overall incidence of anaphylaxis (self-reported) was 0.8% among adolescents. One third of adolescents had access to healthcare during an anaphylactic reaction. Among those who experienced an anaphylactic reaction, 67% had a prescription for an epinephrine auto-injector (e.g., an EpiPen) and 15% received adrenaline treatment during the reaction. Factors associated with an anaphylactic reaction at the age of 16 were: reactions to food at the age of 1–2 (OR: 17.7; 95% CI: 6.91–45.2); sensitization at the age of 4 (OR: 20.9; 95% CI: 6.8–64); polysensitization; having eczema or asthma	Low demand for medical assistance; limited use of adrenaline; lack of awareness of food risks.	Diagnoses of anaphylaxis were based in part on questionnaires completed by parents or participants, without clinical confirmation; the study was conducted in an urban Swedish cohort, so the results may not be generalisable to populations in other countries	3

**Table A5:** Randomized controlled trials included in the present Good Clinical Practice Report on anaphylaxis (Note: CS = cross-sectional study; CC = case–control study; P = prospective design; R = retrospective design, S = single centre, M = multicenter).

Authors	Year	Objectives	Population	Outcome	Results (Quantitative Elements)	Results (Description)	Pico
Umasunthar T. et al. [25]	2015	To evaluate the ability of patients and parents of children with food allergies to recognize anaphylaxis and administer adrenaline auto-injectors (AAIs) appropriately during a simulated emergency.	Children with food allergies were recruited from allergy clinics in the UK (St Mary’s Hospital and Imperial College, both in London).	Administering adrenaline properly via an auto-injector during a simulated anaphylaxis scenario.	After six weeks, 42% of Anapen users (30 out of 71) and 43% of EpiPen users (31 out of 73) successfully administered adrenaline in a simulated anaphylaxis scenario. Success rates at one year were similar to those at six weeks. A study on device switching after one year of follow-up without retraining found that success rates were lower for those who switched from one device to another with a different operating mechanism than for those who switched from one device to another with a similar operating mechanism.	The effectiveness of self-administering adrenaline depends heavily on the type of device used. Auto-injectors equipped with voice instructions can significantly improve the likelihood of correct administration. Adequate and ongoing training is necessary to minimize errors.	1
Kukkonen et al. [44]	2015	To assess the association between sensitization to specific peanut components and the severity of allergic reactions in a pediatric population	Children aged 6–18 years suspected of having a peanut allergy and undergoing an oral challenge.	Measuring sIgE against peanut components as a diagnostic tool for predicting severe allergic reactions.	Of the 69 patients who tested positive (68%), 36% experienced severe reactions, 52% experienced moderate reactions and 12% experienced mild reactions. Thirty-eight people (37%) received adrenaline during the challenge. Ara h6 was the best indicator for discriminating between moderate and severe allergy, with an area under the curve (AUC) of 0.98 (95% CI: 0.96–1.00), while Ara h8 showed no diagnostic value with an AUC of 0.42 (95% CI: 0.30–0.52). Combining Ara h2 and Ara h6 enabled all severe reactions to be identified, even at low allergen doses.	Using Component-Resolved Diagnostics (CRD) that focus on Ara h6, or combining them with Ara h2, can significantly reduce the need for oral challenge tests (OFTs), thereby improving the accuracy of diagnoses of severe peanut allergies.	4
Van Veen et al. [45]	2016	To evaluate the diagnostic effectiveness of component-resolved (CRD) tests and establish whether they could replace oral food challenge tests.	Children with suspected peanut allergy, recruited between 2012 and 2013 at Reinier de Graaf Hospital in Delft, the Netherlands.	Diagnostic utility of CRD (component-resolved) tests.	The double-blind, placebo-controlled food challenge with peanuts produced a positive result in 33 children (53%). No correlation was found between the trigger dose and the severity of the reaction. However, a statistically significant correlation was found between the skin prick test, peanut-specific IgE, Ara h 1, Ara h 2 and Ara h 6, and the outcome of the food challenge test in terms of positivity or negativity (*p* < 0.001). No correlation was found between specific IgE for Ara h 3, Ara h 8 and Ara h 9, and the clinical outcome of the food challenge test.	Molecular diagnostics (also known as component-resolved diagnostics) are not superior to traditional tests such as extract and SPT, and they cannot replace the gold standard of DBPCFC, particularly when it comes to measuring reaction dose or clinical severity of allergy.	4

**Table A6:** Summary of secondary studies (i.e., systematic reviews, narrative reviews, meta-analyses) considered in order to gather the present evidence on anaphylaxis.

Authors	Year	Systematic Review with/Without Meta-Analysis	Objectives	Population	Outcome	Results (Quantitative Elements)	Results (Description)	Pico
Sim M. et al. [28]	2025	SR without MA	To provide a critical evaluation of the effectiveness of adrenaline auto-injectors in preventing fatal anaphylactic outcomes.	Adults and children	The effectiveness of adrenaline auto-injectors (AAIs) in preventing fatal anaphylaxis. The pharmacokinetics and pharmacodynamics of intramuscular adrenaline. Limitations and delays in the use of AAIs. A comparison with intravenous adrenaline. The need for alternative methods of administration.	Studies of animal physiology have shown that intravenous infusions of adrenaline at doses of 0.05–0.5 µg/kg/min for one to two hours (approximately 10 µg/kg in total) are effective in treating severe anaphylaxis, ensuring stable and prolonged plasma levels. In contrast, intramuscular injections, including auto-injectors (AIs), produce short and variable adrenaline plasma peaks (100–500 pg/mL) and a less predictable clinical response.	According to current guidelines, IM adrenaline via an auto-injector remains the recommended first-line treatment, but this may not be sufficient in the most critical cases. In such situations, the authors suggest a more effective approach: continuous, controlled intravenous infusions of adrenaline in combination with supportive fluid therapy. However, implementing this strategy in pre-hospital or home settings still presents significant safety and feasibility challenges.	1
Martelli A. et al. [43]	2020	SR without MA	To provide a summary of recent advances in the epidemiology, diagnosis and management of anaphylaxis, paying particular attention to pediatric patients.	Adults and children	New insights into the epidemiology and triggers of anaphylaxis; advances in diagnosis and biomarkers; updates in treatment approaches and guidelines; attention to differences in the pediatric population	In children, food remains the leading cause of anaphylaxis, while in adults, medications and insect stings are more common. Biomarkers such as tryptase have limited diagnostic utility, particularly in children. Timely administration of intramuscular epinephrine remains the cornerstone of treatment. New approaches include personalized action plans, education, and emerging biological therapies (e.g., omalizumab). It is important to recognize biphasic reactions and comorbidities (e.g., asthma).	Effective management of anaphylaxis requires prompt diagnosis, based on an immediate assessment of the patient’s airways, breathing, circulation, and mental state. Treatment must include the immediate intramuscular administration of adrenaline, which is recognized as the essential first-line therapy. Particular attention should be paid to preventing biphasic reactions, which can occur even after the initial symptoms have resolved. Upon discharge, each child must receive a prescription for an adrenaline auto-injector accompanied by detailed instructions on its correct use. Families must also be provided with a personalized action plan, and the school and wider community must be adequately informed and trained to ensure a rapid and safe response in the event of an anaphylactic emergency.	1
Navalpakam et al. [24]	2013	SR without MA	To providing a comprehensive resource for allergists, focusing on the long-term management of anaphylaxis	Adults and children	Long-term management of anaphylaxis by allergists	A specialist allergy assessment is needed for children after an episode of anaphylaxis to identify triggers and establish a personalized plan. Education should focus on patients at higher risk of anaphylaxis recurrence, such as those with a history of severe symptoms or anaphylaxis, those with a trigger of peanuts and/or nuts, those with a history of asthma and those who are female. Patient counselling should involve providing individualized action plans and discussing the correct use, storage and safety of epinephrine auto-injectors.	The article emphasizes the importance of long-term anaphylaxis management, particularly in an outpatient setting. It highlights the central role of allergists in identifying the cause of anaphylactic reactions and comorbidities, such as asthma or mast cell disorders. They are also responsible for personalizing treatment and reducing the risk of recurrence. Great attention is paid to educating patients and caregivers: they must know how to recognize symptoms early, administer auto-injectable adrenaline correctly, and manage emergency situations. For this reason, the article recommends individualized action plans, medical identification tools (such as bracelets or cards) and careful follow-up.	1
Dribin et al. [19]	2025	Consensus statement	To develop a standardized, internationally accepted definition of anaphylaxis, alongside an educational overview and clinical support tools.	Adults and children	To improve the recognition, diagnosis and management of anaphylaxis in different clinical settings by replacing existing inconsistent criteria.	Anaphylaxis is defined as a severe, potentially life-threatening allergic reaction that affects the skin, mucous membranes, respiratory system, cardiovascular system, and gastrointestinal tract. Severe anaphylaxis can affect breathing and/or the heart, even in the absence of skin symptoms. The diagnostic criteria are: -unknown exposure (a probable diagnosis is made if skin and/or mucous membrane symptoms and respiratory and/or cardiovascular involvement occur rapidly); -known exposure (a rapid onset of at least two signs from the skin, respiratory, cardiovascular and/or gastrointestinal systems, or respiratory and/or cardiovascular involvement alone). The treatment of choice is adrenaline (IM 0.01 mg/kg, max. 0.5 mg), which can be repeated every 5–15 min. Administer immediately, even if the criteria are not fully met. Systemic signs include skin, mucous membrane, respiratory and cardiovascular involvement, and gastrointestinal symptoms. In children, examples include “lip licking” in infants.	The GA^2^LEN 2024 consensus provides a new definition of anaphylaxis (93.5% agreement), an educational overview (97.8%) and a clinical tool (93.5%) to promote clarity in diagnosis and treatment. This intuitive tool is also suitable for pediatric settings with limited experience and encourages the timely use of adrenaline, which can improve clinical outcomes and research consistency globally.	1
Deschildre et al. [26]	2024	Consensus statement	To improve the knowledge and confidence of school staff in recognizing and managing food allergies and severe allergic reactions. Ensure that there are written emergency action plans for each pupil with a food allergy and that staff are trained in the safe administration of adrenaline.	Children	A standardized management framework, improved safety in school environments, emergency preparedness and education and awareness-raising.	It proposes four pillars to ensure safe schools for students with food allergies: (1) staff training through questionnaires and regular courses on allergies, symptoms, anaphylaxis, auto-injectors and psycho-emotional aspects; prevention with clear policies on spaces, meals, cleaning, allergen labelling; (2) identification of at-risk students without stigmatization and avoidance of general bans; (3) emergency preparedness with up-to-date written plans, availability of auto-injectors for general use, adequate storage and accessibility, and legal protection for staff; (4) inclusive culture with adapted educational materials, anti-bullying activities, regular policy review, and a designated coordinator with feedback from students.	The GA^2^LEN–EFA 2024 consensus sets out minimum guidelines, agreed by over 80 experts, for improving the management of food allergies and anaphylaxis in schools. The four intervention areas propose a flexible, integrated strategy adaptable to different regulatory contexts, aiming to ensure safety, autonomy, and educational continuity, and transform schools into environments that are truly protected and aware.	1
Anagnostou, K. et al. [22]	2018	SR without MA	To synthesize current knowledge to support clinicians in the diagnosis, management and prevention of pediatric anaphylaxis.	Patients aged <18 years	Current knowledge on diagnosing, managing and preventing pediatric anaphylaxis.	Annual incidence: 50–112 episodes per 100,000 people. Rates are higher in the first two years of life (almost three times higher than in other age groups). Anaphylaxis is more common in males up to the age of 10–15; in females, the rate tends to increase after this age. Foods are the main trigger in children, particularly eggs, cow’s milk, and nuts. Risk factors for severe outcomes include uncontrolled asthma, previous biphasic or protracted episodes, repeated administration of epinephrine, wheezing, hypotension, and pharyngeal edema.	The number of cases of anaphylaxis in children is increasing, particularly in the early years of life and among males. Food is the main trigger. Effective management relies on prompt diagnosis, the correct use of auto-injectable adrenaline, the recognition of risk factors (such as uncontrolled asthma), and post-crisis observation categorized according to individual risk. In addition, it is very important that patients and families receive regular training on how to identify anaphylactic episodes and respond appropriately, and this should form part of routine management.	1
Ball et al. [21]	2023	SR without MA	To analyze the changes to the 2021 guidelines and provide an assessment of their practical implications for anaphylaxis treatment, particularly in the pediatric population.	Adults and children	Guidelines for the management of anaphylactic emergencies, with particular attention to the pediatric population	If airway, breathing or circulation problems persist after 5 min, repeat the intramuscular epinephrine injection. The new recommended dose of epinephrine for children under 6 months is 100–150 μg (1 in 1000). Corticosteroids are no longer recommended for the emergency treatment of anaphylaxis. Children under 16 years of age who have received emergency treatment for suspected anaphylaxis should be admitted to hospital. If an auto-injector is required for a child under 12 months of age, it should only be prescribed by a pediatric allergist. In children under 16 years of age after a suspected anaphylactic reaction, tryptase testing is useful if the cause is thought to be related to venom, medication or idiopathic factors.	Analysis of the 2021 RCUK guidelines confirms that the primary treatment for anaphylaxis is intramuscular adrenaline, with a strong recommendation to administer a second dose after five minutes if clinical signs persist. The routine use of corticosteroids has been discontinued, with antihistamines now only recommended for residual skin symptoms. New recommendations include the strategic use of intravenous (IV) fluids, the introduction of a protocol for refractory anaphylaxis involving adrenaline infusion, and a stratified approach to post-event observation. Adopting the GRADE framework ensures transparency and methodological robustness. These changes improve operational clarity, potentially improving survival rates and post-anaphylactic management in healthcare settings.	1 and 2
Brockow, K. et al. [78]	2021	SR without MA	To summarize and analyze the available evidence regarding symptoms of mast cell activation and anaphylactic reactions in children with mastocytosis.	Children with cutaneous mastocytosis	To identify the risk factors, prevalence, clinical manifestations, management strategies and treatment of anaphylaxis in children with mastocytosis.	The incidence of anaphylaxis in children with cutaneous mastocytosis is 1–9%. Risk factors include involvement of more than 90% of the body’s surface area and elevated serum tryptase levels (above 20 ng/mL). Baseline serum tryptase levels should be measured, as very high levels (above 100 ng/mL) may indicate a high mast cell burden and/or secondary mastocytosis. Serum tryptase levels are usually normal or slightly elevated in CM but elevated in most patients with SM and DCM. In children with tryptase levels above 10 µg/L and severe MC symptoms, analyzing extra copies of the alpha-tryptase gene (TPSAB1) can help to understand the genetic basis of these symptoms.	Although mastocytosis in children carries a risk of mast cell-mediated events, anaphylaxis is less common than in adults. Children with severe skin symptoms and high tryptase levels are more likely to experience anaphylaxis and require closer clinical monitoring. A baseline tryptase measurement should be taken 24–48 h after symptoms have resolved to assess risk. Further genetic testing is recommended in cases where tryptase levels are greater than 10 μg/L and there are severe mast cell-mediated symptoms.	2
Wang J. et al. [36]	2024	SR without MA	To update and clarify the clinical recommendations for diagnosing, managing and preventing anaphylaxis in daily practice for adults and children.	Adults and children	Update on the diagnosis, management and prevention of anaphylaxis	The current anaphylaxis criteria can be applied to infants and children to determine whether an allergic reaction is anaphylaxis. After prompt self-administration of epinephrine, hospital admission is not necessary if the patient responds completely and sustainably. Take serum tryptase during an acute event (within two hours of symptom onset) and at a separate time when the patient is in normal health to help diagnose anaphylaxis. Do not rely solely on serum tryptase levels to diagnose underlying mastocytosis. Perform skin tests (percutaneous and intradermal) and/or specific in vitro IgE tests four to six weeks after the event for all potential pharmacological and non-pharmacological agents used during the perioperative period.	The introduction of new, more accurate diagnostic criteria for anaphylaxis includes new biomarkers, such as tryptase. While the use of epinephrine remains central, it is no longer the only method of diagnosis. Management is personalized according to the patient’s age, the clinical context, and their response to treatment, allowing for greater flexibility in the activation of emergency services. Empowering patients is central to this approach, achieved through targeted counselling and shared decision-making.	2
Giannetti A. et al. [35]	2021	SR without MA	To provide an overview of mast cell activation disorders (MCAD), including classification, pathophysiology, clinical manifestations, diagnostic criteria, and treatment options.	Children	Classification and diagnostic criteria of MCAD; pathophysiological mechanisms of mast cell activation; clinical presentation and differential diagnosis; therapeutic approaches and management strategies	Symptoms are caused by the release of mediators, such as histamine, which can affect multiple systems, including the skin, gastrointestinal system, respiratory system and cardiovascular system. There is currently no consensus on specific biomarkers for MCAS. Diagnosis is based on clinical symptoms, elevated serum tryptase levels or other mediator levels, and response to anti-mediator therapy. Treatment includes avoiding triggers, taking antihistamines and mast cell stabilizers, and using corticosteroids. In severe cases, biological drugs such as omalizumab may be required. As mast cell activation syndrome can mimic other conditions, a multidisciplinary approach is essential.	Mast cell activation syndrome (MCAS) is characterized by normal mast cell density, but with functional hyperactivity. In contrast, mastocytosis is characterised by an abnormal accumulation of mast cells in tissues. Currently, the lack of specific and shared biomarkers complicates diagnosis, which is primarily based on clinical evaluation and laboratory tests. Given the absence of definitive treatments, therapeutic management remains predominantly symptomatic, with the aim of controlling signs and symptoms. This review therefore emphasizes the importance of accurate clinical investigation and a better understanding of the characteristics of mast cell activation disorders, with the aim of improving diagnosis and optimizing patient care.	2
Calvani M. et al. [40]	2020	SR without MA	To provide an up-to-date overview of food allergies, focusing on their pathogenesis, clinical characteristics, diagnostic tools, preventive strategies and therapeutic management, particularly in children.	Adults and children	The immunological mechanisms underlying food allergies, advances in diagnostic approaches, and prevention strategies, particularly the early introduction of allergens, as well as current and emerging treatments.	A diagnosis requires a combination of clinical history, specific IgE testing, skin testing and oral food challenge testing. Preventive strategies now support the early introduction of allergenic foods (e.g., peanuts and eggs) in infants at risk. Management includes elimination diets, emergency plans (e.g., epinephrine auto-injectors) and emerging therapies such as oral immunotherapy (OIT). Patient and caregiver education are essential for safety and quality of life.	Food allergy is a condition that is becoming increasingly prevalent, particularly among children, and requires an integrated approach based on early diagnosis, targeted prevention, and personalized treatment strategies. In terms of diagnosis, Component-Resolved Diagnostics (CRD) are emerging as a more accurate tool than traditional tests. They can guide clinical decisions and reducing the need for oral provocation tests. Regarding prevention, the most recent evidence suggests that allergens should be introduced early in at-risk infants, challenging the traditional approach of avoidance. Finally, management strategies are evolving beyond dietary exclusion thanks to emerging therapies such as oral immunotherapy, which show promise in modifying the natural course of the disease.	3
Yue, D. et al. [39]	2018	SR without MA	To summarize the recent advances in understanding, diagnosing and managing food allergies and anaphylaxis, including the latest prevention and treatment strategies.	Adults and children	Diagnosis, management, prevention and treatment strategies for food allergies and anaphylaxis	The diagnosis is based on detailed clinical history; skin tests (SPT); specific IgE dosage; basophil activation tests (BAT) in some cases. Massive release of mediators (histamine, tryptase and PAF) generates the clinical response. Reduced activity of the PAF-acetylhydrolase enzyme is associated with more severe forms. Oral food challenge (OFC) remains the gold standard. Underuse of epinephrine is still common, even in healthcare settings. Emerging evidence (such as that from the LEAP study) supports the early introduction of allergenic foods in high-risk children.	Yue et al. (2018) [39] propose an updated view of allergy-related anaphylaxis. They emphasize that, although most cases are IgE-mediated, anaphylaxis can also have alternative mechanisms with similar clinical implications. The clinical presentation is determined by the massive release of inflammatory and immunological mediators (including PAF). The severity is partly dependent on the activity of the PAF-acetylhydrolase enzyme. The diagnosis remains essentially clinical. However, allergy tests (e.g., skin prick tests, specific IgE assays and oral provocation tests) are essential for identifying the responsible allergen. This is particularly important for preventing recurrence. IM adrenaline is the cornerstone of acute treatment. In the long term, the approach revolves around allergen avoidance. In the future, food immunotherapy protocols may also be used as a promising therapeutic option.	3
Turner et al. [41]	2022	SR with MA	To summarize the most recent evidence on the risk factors for severe allergic reactions to food.	Patients aged under 40 years with an IgE-mediated food allergy or FPIES.	To identify modifiable risk factors in patients with severe food allergies.	Mortality from food anaphylaxis is estimated at 1.81 per million person-years. Near-fatal reactions are approximately ten times more common than deaths but are still rare. Non-predictive factors include previous anaphylaxis, asthma and baseline IgE sensitization. Established risk factors include adolescent/young adult age (12–30 years) and delays in treatment (e.g., epinephrine). Emerging or uncertain factors include specific allergenic components, risk behaviour, concomitant medications, dose and physical exercise.	Currently, there are no reliable clinical or laboratory markers that can accurately predict an individual’s risk of experiencing a severe or fatal reaction to food. However, adolescence and young adulthood, as well as delayed identification and treatment, are well-established risk factors for severe reactions.	3 and 4
Kattan J. D. and Sicherer S. H. [42]	2015	SR without MA	To analyze emerging approaches and future prospects for diagnosing food allergies.	Adults and children	To analyze emerging approaches and future prospects for diagnosing food allergies.	Many individuals who test positive for Skin prick Test (SPT) or sIgE are tolerant to food in oral challenge tests (OCT). For example, only 22% of individuals sensitized to peanuts were clinically allergic. OCT is expensive and labour-intensive, but it is the gold standard and safe in clinical settings (only 1.7% of individuals required epinephrine). CRDs (e.g., Ara h2 and Cor a9/14) offer greater specificity than traditional tests, but often lower sensitivity. Emerging tests, such as epitope binding, can differentiate between tolerant and persistent individuals (e.g., milk) and predict reactivity (e.g., shrimp). T-cell responses can potentially distinguish between individuals who are sensitised but asymptomatic and those who are allergic and symptomatic. Basophil activation is a promising method for predicting tolerance to cooked foods (e.g., cooked milk) and potentially the severity of reactions, but it requires flow cytometry and extensive validation. PAF and PAF AH correlate with anaphylaxis severity, with low PAF AH and high PAF being associated with severe and fatal reactions.	Although traditional tests (SPT and sIgE) are useful as screening tools, they have limitations in terms of specificity and predictability. Sensitization often does not coincide with clinical allergy. Oral food challenge (OFC) remains the gold standard for confirming a diagnosis and has a high rate of negative reactions with manageable risks. Methods such as CRD improve specificity but reduce sensitivity. Emerging tests (epitope binding, basophil/T-cell reactions and PAF) show significant potential for future diagnostics but are currently limited by technical complexity or poor clinical validation.	4
Martelli A. et al. [43]	2021	SR without MA	To explore the role of component-resolved diagnosis (CRD) in improving the accuracy of food allergy diagnosis and risk assessment, with a particular focus on its clinical application in identifying allergenic protein components.	Adults and children	To provide an overview of CRD technology and its clinical utility and covers the identification of specific allergenic components related to severity and persistence, the differentiation between primary sensitization and cross-reactivity, and the use of CRD in predicting the risk of systemic reactions.	CRD enables the precise identification of IgE sensitization to specific protein components, such as storage proteins, lipid transfer proteins and profilins. It can distinguish between genuine food allergies and cross-reactions, such as pollen-food syndrome. Some components, such as Ara h 2 in peanut allergy, are strongly associated with severe reactions. CRD improves the stratification of individual risk, guiding decisions on dietary avoidance and immunotherapy. It is particularly useful in complex cases and for determining when an oral food challenge is necessary.	Molecular diagnosis (CRD) represents a significant advance in the management of food allergies, thanks to its ability to refine diagnosis, assess individual risk profiles and reduce the use of invasive tests. However, its clinical applications continue to be limited by the lack of established diagnostic standards and variability between studies. Until robust, standardized tests are available, CRD should be used as a complementary tool, always integrated with clinical history and, if necessary, confirmed by oral challenge tests.	4
Cardona V. et al. [2]	2020	Guideline	To ensure that the guidelines are aligned with the current state of knowledge regarding anaphylaxis management.	Adults and children	Guidelines for managing anaphylactic emergencies	The guidelines recommend intramuscular adrenaline (0.01 mg/kg, up to a maximum of 0.5 mg) in the anterolateral thigh as the initial treatment. This should be administered immediately and repeated every 5–15 min if necessary. Patients with anaphylaxis should be referred to an allergist/immunologist for advice on identifying and preventing triggers. During acute anaphylaxis, serum tryptase levels increase from 15 min to three hours after the onset of symptoms, peaking between one and two hours afterwards. It is recommended that baseline serum tryptase levels are assessed at least 24 h after the symptoms of anaphylaxis have resolved, even when the concentration of tryptase during the episode remains within normal limits. Allergic sensitization is diagnosed using skin tests, allergen-specific serum IgE and provocation tests.	The WAO 2020 guidelines update the management of anaphylaxis by emphasizing the importance of rapid diagnosis and the optimal use of intramuscular adrenaline, as well as structured long-term interventions such as the prescription of auto-injectors, education involving an action plan, and the involvement of specialists. The guidelines also highlight disparities in access to auto-injectors and propose strategies to overcome these barriers. The guidelines also highlight gaps in education, post-emergency management and epidemiological data collection.	1, 2, 3 and 4
Santos et al. [3]	2023	Guideline	To provide practical recommendations for the diagnosis of severe IgE-mediated food allergy	Adults and children	Practical recommendations for the diagnosis of severe IgE-mediated food allergy	Peanut (Ara h 2, CRD): Sensitivity: 95%; Specificity: 61%. High identification capacity, but risk of false positives. Cow’s milk (whole allergen sIgE): Sensitivity: 53%; Specificity: 88%. Useful for ruling out allergy, but less so for confirming it. Egg (whole sIgE allergen): sensitivity 92%, specificity 58% = high sensitivity with a risk of overdiagnosis. Wheat and soy (whole sIgE allergen): Sensitivity and specificity are between 55% and 73%, indicating less defined diagnostic performance.	Compared to SPT and total sIgE, specific IgE tests, particularly those for peanut allergens such as Ara h 2, improve diagnostic accuracy. However, high sensitivity and variable specificity require clinical interpretation and confirmation with oral food challenge (OFC) to avoid misdiagnosis and unnecessary dietary restrictions. The variability between foods emphasizes the importance of a personalized approach that integrates sIgE, CRD, SPT and BAT, with OFC serving as the gold standard for diagnosis when necessary.	3 and 4

**Table A7:** Original studies included in the present Good Clinical Practice Report on drug allergy (Note: CS = cross-sectional study; CC = case–control study; P = prospective design; R = retrospective design, S = single centre, M = multicenter).

Authors	Year	Type of Study	Objectives	Population (General Characteristics)	Sample Size	Outcome	Results (Quantitative Elements)	Results (Description)	Limitations	Pico
Moral et al. [53]	2022	CS, R, M	To describe the clinical characteristics of pediatric patients with a presumed positive or inconclusive drug provocation test (DPT) for hypersensitivity to beta-lactam antibiotics (BLA), to evaluate the decision to repeat the DPT, and describe the outcome.	Patients (<15 years of age) who attended one of the six participating pediatric allergy clinics between January 2017 and December 2019.	439 children underwent beta-lactam challenge testing; 26 of them (5.9%) with presumed positive or inconclusive results were included in the study.	Interpretation of initial DPT results, decision to repeat the DPT, and outcome of the repeated DPT	Only 5.9% of patients evaluated for suspected BLA allergy had a potentially positive DPT. However, more than half of patients with a positive or inconclusive DPT-1 tolerated BLA during DPT-2, most of which was performed within 6 months of DPT-1.	This study concludes that a DPT positive for BLA should preferably be confirmed with a second DPT, to be performed within a few weeks or months.	Retrospective study, small sample size and heterogeneous practices in participating centres.	1
Goh et al. [49]	2021	C, R, S	To evaluate the results of oral provocation tests (OPT) in children with suspected hypersensitivity reactions to beta-lactam antibiotics	Children (<18 years) treated at KK Women’s and Children’s Hospital, Singapore, between August 2016 and December 2017	120	The proportion of negative OPT results; the frequency of mild adverse reactions; the diagnostic accuracy of OPT	93% of OPTs were negative (110/118): 92% negative for the penicillin group (96/104), 100% negative for cephalosporins (14/14).	All reactions were mild	Retrospective design. Limited assessment of hypersensitivity to clavulanic acid. Small sample size for some drug groups.	1
Labrosse et al. [52]	2020	C, P, S	To evaluate the sensitivity and specificity of the double-blind penicillin skin test (PST) with penicilloyl-polylysine (PPL) and benzylpenicillin (BP) in predicting reactions to oral provocation test with amoxicillin in children with a history of non-life-threatening penicillin allergy	Children aged between 0.7 and 18.1 years with a history of non-severe penicillin allergy, referred to a tertiary allergy centre.	158	Sensitivity and specificity of PST in predicting reactions to oral provocation with amoxicillin; positive and negative predictive values; proportion of patients with positive PST and subsequent reactions	Sensitivity: 20% (95% CI: 0.5–71.6%). Specificity: 90% (95% CI: 84.4–94.4%). Positive predictive value: 6.3% (95% CI: 0.4–26.3%)	3.2% of participants had an immediate or accelerated reaction to the amoxicillin challenge; all reactions were mild and resolved with antihistamines.	Low incidence of events, resulting in wide confidence intervals. Exclusion of accelerated reactions led to an estimated sensitivity of 33% (95% CI: 0.8–90.5%). Lack of intradermal testing with amoxicillin due to its unavailability in Canada. Possible variability in skin test results that may affect reliability.	1
Mill et al. [57]	2016	C, P, S	To evaluate the diagnostic accuracy of a graded oral challenge (OC) in diagnosing both immediate and non-immediate allergic reactions to amoxicillin in children	Children (between 1 and 3.9 years old) with suspected reaction to amoxicillin	818	The proportion of children who experienced immediate and non-immediate reactions to graded OC. Measures of the diagnostic accuracy (sensitivity, specificity and positive and negative predictive values) of graded OC associated with immediate and non-immediate reactions	94.1% of children tolerated the graduated OC. 2.1% had immediate reactions (all mild). 3.8% had non-immediate reactions (all mild). The graduated OC showed 100% specificity, 100% positive predictive value, and 89.1% negative predictive value.	Factors associated with immediate reactions: history of reaction within 5 min of exposure. Factors associated with non-immediate reactions: rash lasting more than 7 days and family (parental) history of drug allergy.	It is impossible to determine the sensitivity of the graded OC due to the study design. There is a possibility of classification bias due to reliance on parental reports. The results are limited in their generalisability to populations other than the one studied.	1

**Table A8:** Summary of secondary studies (i.e., systematic reviews, narrative reviews, meta-analyses) considered in order to gather the present evidence on drug allergy.

Authors	Year	Systematic Review with/Without Meta-Analysis	Objectives	Population	Outcome	Results (Quantitative Elements)	Results (Description)	Pico
Paño-Pardo et al. [48]	2023	Consensus document	To formulate evidence-based recommendations aimed at improving the management of patients with suspected or confirmed antibiotic allergy, as well as standardizing the approach of both clinicians in prescribing antimicrobial agents to patients labelled as allergic and allergists in confirming or excluding the label of antibiotic allergy.	These guidelines are not limited to patients of a specific gender or age group. Instead, they take a comprehensive approach that includes allergies to all classes of antibiotics.	Epidemiology of antibiotic allergies; Risk assessment in patients labelled as allergic to antibiotics; Assessment of patients with suspected antibiotic allergies through additional testing; Choice of antibiotic in patients with reported allergies to penicillin or cephalosporins.	Eleven recommendations were formulated, all with a strength rating of A: solid evidence supporting a recommendation for or against use.	Epidemiology of antibiotic allergies; Risk assessment in patients labelled as allergic to antibiotics; Assessment of patients with suspected antibiotic allergies through additional testing; Choice of antibiotic in patients with reported allergies to penicillin or cephalosporins.	1
Sousa-Pinto et al. [46]	2021	SR with MA	To evaluate the effectiveness of skin tests and the quantification of specific IgE in diagnosing patients with allergies to penicillin or other β-lactams.	Patients with reported allergy to penicillin or β-lactams, in whom the results of the DPT were compared with those of skin tests (prick test and/or intradermal test) and/or with the quantification of specific IgE (sIgE).	Sensitivity and specificity of skin tests and specific IgE quantification; predictive values (positive and negative) of these tests	Skin tests: sensitivity 30.7%, specificity 96.8%; specific IgE: sensitivity 19.3%, specificity 97.4%	Both tests show low sensitivity and high specificity.	1
Felix et al. [50]	2020	SR	To evaluate the safety and efficacy of direct OPT in the diagnosis of beta-lactam allergy in children with mild skin reactions.	Children with a history of mild, immediate and non-immediate skin reactions to beta-lactams	Proportion of negative OPT results; frequency of mild adverse reactions; safety of direct OPT		High proportion of negative results in OPT; most patients tolerated OPT without serious reactions.	1
Mori et al. [54]	2019	Reviews	To provide a comprehensive overview of the management of suspected reactions to antibiotics in children, including diagnostic approaches and treatment strategies.	Children suspected of having an allergic reaction to antibiotics.	Diagnostic strategies for antibiotic hypersensitivity in children; management approaches for suspected antibiotic reactions	Emphasis on the importance of a detailed clinical history in the diagnosis of antibiotic hypersensitivity; discussion of various diagnostic tests, including skin tests and drug challenge tests; recommendations for management strategies based on the type of reaction and the suspected antibiotic.	Diagnostic strategies for antibiotic hypersensitivity in children; management approaches for suspected antibiotic reactions	1
Calamelli et al. [55]	2019	Review	Provide a practical approach to managing children who have experienced adverse reactions to antibiotics, emphasizing the importance of accurate diagnosis and appropriate management strategies.	Children who have experienced an adverse reaction to antibiotics	Overview of diagnostic tools and procedures used to identify antibiotic allergies in pediatric patients; guidance on how to manage and treat children with suspected antibiotic-related allergic reactions.	It emphasizes the key role of taking an accurate clinical history to distinguish true allergic reactions from non-allergic side effects; it describes the use of skin tests and oral provocation as essential methods for confirming or ruling out an allergy; it offers practical recommendations tailored to the type and severity of the reaction, with the aim of unnecessary avoidance of antibiotics and optimizing treatment options.	Overview of diagnostic tools and procedures used to identify antibiotic allergies in pediatric patients; guidance on how to manage and treat children with suspected antibiotic-related allergic reactions.	1
Romano et al. [4]	2020	Position paper	To update and to standardize the diagnostic approach to beta-lactam hypersensitivity, integrating evidence-based guidelines and expert consensus.		Development of algorithms for risk stratification. Standardization of protocols for skin testing (ST) and DPT. Recommendations for the administration of alternative beta-lactams in allergic individuals.	Introduction of risk stratification based on morphology, timing and severity of the reaction. Emphasis on the use of ST and DPT in the diagnosis of hypersensitivity to beta-lactams. Recommendations for the administration of alternative beta-lactams in allergic individuals.	Development of algorithms for risk stratification. Standardization of protocols for ST and DPT. Recommendations for the administration of alternative beta-lactams in allergic individuals.	1
Caffarelli et al. [47]	2018	Position paper	To provide guidelines for managing suspected allergies to penicillin and β-lactam antibiotics, including diagnostic and therapeutic strategies.		Recommendations for performing DPTs in children with suspected penicillin/β-lactam antibiotic allergy. Guidelines for managing immediate and delayed reactions to penicillin/β-lactam antibiotics in pediatric patients.	The DPT is identified as the most reliable method for confirming or ruling out β-lactam allergy in children. The document provides detailed, step-by-step protocols for performing DPTs safely, including dosing schedules and monitoring measures. The critical role of a thorough medical history and proper risk assessment in determining, when DPT, is appropriate is emphasized. The guidelines also offer strategies for managing any allergic reactions that may occur during or after the test.	Recommendations for performing DPTs in children with suspected penicillin/β-lactam antibiotic allergy. Guidelines for managing immediate and delayed reactions to penicillin/β-lactam antibiotics in pediatric patients.	1
Brockow et al. [56]	2025	Position paper	Provide guidelines for managing suspected allergies to penicillin and β-lactam antibiotics, including diagnostic and therapeutic strategies.		Recommendations for performing DPTs in children with suspected penicillin/β-lactam antibiotic allergy. Guidelines for managing immediate and delayed reactions to penicillin/β-lactam antibiotics in pediatric patients.	The DPT is identified as the most reliable method for confirming or ruling out β-lactam allergy in children. The document provides detailed, step-by-step protocols for performing DPTs safely, including dosing schedules and monitoring measures. The critical role of a thorough medical history and proper risk assessment in determining when DPT is appropriate is emphasized. The guidelines also offer strategies for managing any allergic reactions that may occur during or after the test.	Recommendations for performing DPTs in children with suspected penicillin/β-lactam antibiotic allergy. Guidelines for managing immediate and delayed reactions to penicillin/β-lactam antibiotics in pediatric patients.	1

**Table A9:** Original studies included in the present Good Clinical Practice Report on hymenoptera venom allergy (Note: CS = cross-sectional study; CC = case–control study; P = prospective design; R = retrospective design, S = single centre, M = multicenter).

Authors	Year	Type of Study	Objectives	Population (General Characteristics)	Sample Size	Outcome	Results (Quantitative Elements)	Results (Description)	Limitations	Pico
Worm et al. [60]	2014	CS, P, M	The aim of the NORA study was to collect and analyze prospective data on cases of anaphylaxis treated in European specialist centres in order to describe their causes, clinical characteristics, therapeutic management and differences between pediatric and adult populations.	The study analyzed a very large and diverse population (26.7% of cases under the age of 18, 73.3% adults) of patients suffering from severe allergic reactions (anaphylaxis) in several European countries.	3333 cases of anaphylaxis collected via an online questionnaire based on clinical and diagnostic data between June 2011 and March 2014.	The study highlighted significant differences in the triggers and clinical presentation of anaphylaxis in children and adults. It emphasized the frequent underuse of adrenaline as an emergency treatment and confirmed the need to improve the early identification and management of the condition across Europe.	59 allergy/dermatology/pediatrics centres in 10 European countries. 3333 episodes of anaphylaxis recorded. 26.7% of patients were aged < 18 years; 73.3% were adults. In children, the most frequent cause of anaphylaxis (64.9%) was food; in adults, the most frequent cause (47.4%) was medication. Hymenoptera venom was the cause of anaphylaxis in children in 20.2% of cases. 80.5% of cases occurred within 30 min of exposure; only 6.7% had a delay of more than 4 h (mainly in cases involving drugs). Skin symptoms were present in 84.1% of cases. 34.2% had already had a reaction, usually milder, to the same allergen. Among the treatments administered by professionals as first aid, 60.4% were corticosteroids and 52.8% were antihistamines. Adrenaline was administered at the site of the event in 13.7% of cases due to food and in 27.6% of cases due to hymenoptera venom.	The NORA study described 3333 cases of anaphylaxis in Europe, highlighting how the causes, clinical manifestations and management vary significantly between children and adults. Food is the main cause in children, while drugs and insect venom predominate in adults. Respiratory symptoms are more common in children than in adults, while cardiovascular symptoms are more common in adults. In both children and adults, skin involvement is the predominant clinical manifestation. Despite clinical recommendations, adrenaline has been underused as an emergency treatment, indicating a need for improved medical education and early response to anaphylaxis.	It is not exclusively focused on pediatric populations. It is based on observational and non-experimental data collection from spontaneous reports, which may lead to overestimation or underestimation of the data.	1
Quercia et al. [58]	2014	CS, R, S	To assess the actual prevalence and incidence of reactions to hymenoptera venom in the pediatric population, with particular attention to systemic reactions and extensive local reactions. To estimate the annual incidence of new allergic reactions in that population during the 2 years following the initial survey. To investigate the clinical management of children who had experienced significant reactions.	1035 children aged between 6 and 14 in the town of Cotignola (RA). Study conducted in the town’s state schools.	1035 children	The study results do not confirm recent reports of an increase in the prevalence of hymenoptera venom allergy in children. The study reduces the perception of the risk of severe systemic reactions to hymenoptera stings in children and suggests that the prevalence is much lower than previously estimated, the incidence of new reactions is statistically negligible, clinical management is often inadequate and needs to be improved, especially with regard to allergy diagnosis and the use of immunotherapy.	Of a population of 1035 children, 173 (16.7%) had been stung by hymenopterans at least once. Of these, five (0.5%) had a systemic reaction (SR) and nine (0.9%) had an extensive local reaction (LLR). Only one reaction was severe. Of the 14 subjects with an SR or LLR, five (35.7%) underwent diagnostic evaluation, and one (7.1%) received venom immunotherapy. The incidence of SR was 0.09% in the first year and 0.08% in the second year.	The results of the study suggest that severe reactions to hymenopteran stings in children are rarer than previously estimated, and emphasize the importance of improving the diagnostic and therapeutic pathways for clinically relevant cases.	Observational and non-experimental data collection based on questionnaires, with possible overestimation or underestimation of data. Limited number of reactions observed. Data comes from a single city in Italy and may not be generalizable to other populations or geographical contexts.	1
Graif et al. [79]	2009	CS, R, M	This study aimed to assess the prevalence and severity of allergic reactions to insect stings in children with atopy, compared to those without, to determine whether atopy is a risk factor for a higher incidence and severity of hymenoptera venom reactions.	Israeli teenagers aged between 13 and 14	10,021 teenagers	Children with atopic diseases reported a significantly higher frequency of allergic reactions than non-atopic children. The reactions were more severe in children with atopy.	A total of 10,021 questionnaires were useful for analysis. Among children who reported insect bites (56.3%), the prevalence of current asthma was 6.0%, allergic rhinitis 10.5% and atopic eczema 8.7%, with no significant differences compared to the entire study population. Among children with any of the atopic diseases, 36.9% reported an allergic reaction to insect bites, compared with 24.8% of non-atopic children (*p* < 0.0001). In the multivariate analysis, asthma, allergic rhinitis, and atopic eczema were significant risk factors for allergic reactions of any severity. Children in the atopic group had a significantly higher rate of severe allergic reactions than non-atopic children, as well as relatively higher rates of mild reactions (*p* < 0.0001). Asthmatic patients with severe allergic reactions had more parameters of severe asthma than asthmatic patients with mild or no reactions.	The data showed that more than half of the children (56.3%) had reported at least one insect sting during their lifetime. Among these, a significant proportion developed allergic reactions, classified into three levels: extensive local reactions (LLR), mild systemic reactions (MSR) and severe systemic reactions (SSR). Children with atopic diseases showed a higher frequency of allergic reactions than their non-atopic peers. The severity of reactions was also higher in atopic subjects. In particular, these children reported more intense systemic reactions, such as widespread urticaria, breathing difficulties and extensive edema.	The data were collected through observational and non-experimental methods based on questionnaires. There is a possibility of overestimation or underestimation of the data, and there is a lack of clinical confirmation of atopic conditions. The data were collected in a single country (Israel) and may not be generalizable to other populations or geographical contexts.	2
Tischler et al. [76]	2025	CS, R, S	To assess whether the quantitative ratio of bee (BV) and Vespula (VV) venom specific IgE can identify the responsible insect in patients with dual sensitization and determine whether dual immunotherapy is necessary in cases of dual sensitization to specific components.	patients (adult and pediatric population) with dual sensitization to bee and Vespula venom, confirmed through clinical and serological tests	1069 consecutive patients with suspected insect venom allergy (bee or wasp) and 490 non-allergic subjects, used for statistical comparisons.	A specific IgE ratio ≥ 5:1 is a strong indicator of the ‘culprit’ venom in double-sensitized patients. Double immunotherapy is not necessary in cases where a dominant venom is clearly identified.	Patients with dual sensitization (to both bee and Vespula venom) 459; among these, 239 patients (52.1%) had a specific BV/VV IgE ratio of at least 5:1 (dominance > 5:1 towards one venom). Of these patients, 232 (97.1%) were found to be monoallergic to the dominant venom.	A specific IgE ratio ≥ 5:1 (favouring BV or VV) is a highly reliable indicator of the venom responsible in patients with dual sensitisation. In most cases (97.1%), this parameter allows the culprit to be correctly identified, avoiding unnecessarily prolonged treatment.	Retrospective study based on previously collected clinical data, which may be subject to selection bias and data incompleteness. Data collected at a single centre. Population not exclusively pediatric. Use of an IgE threshold that is not universally standardized. Results not confirmed by provocation tests.	2
Baker et al. [71]	2016	CS, R, S	To evaluate the accuracy with which allergists can identify stinging insects, and analyze their standard procedures for assessing individuals suspected of being hypersensitive to insect stings.	Allergists attending the 2013 American College of Allergy, Asthma, and Immunology conference were invited to participate in the study.	79, of whom 36 were specialist allergists and 43 were non-specialists	The primary outcome is the accuracy of identifying the biting insect, measured using a standardized test, while secondary outcomes concern the clinical diagnostic habits of specialists.	Allergists are collectively more skilled at identifying insects than non-allergists. Overall, the average number of correct answers for non-allergists was 5.4 (2.0) out of a total of 10. This score was significantly lower than that of allergists (6.1 [2.0]; *p* = 0.01) who participated in the study. Most allergists (78.5%) test for all stinging insects and use skin testing (69.5%) as their first test of choice in evaluating individuals with insect hypersensitivity.	Allergists demonstrated a greater ability to correctly identify stinging insects than non-specialists, with an average score of 6.1 out of 10 compared to 5.4 for non-specialists, but even they can confuse some species of stinging insects. Most allergists report testing for all stinging insects when evaluating patients with suspected sting allergy, demonstrating a comprehensive diagnostic approach. Skin testing is the most frequently used initial diagnostic method.	The sample size is limited. The study was conducted at a single location and the sample may not be sufficiently representative.	2
Kokcu Karadag et al. [75]	2024	CS, P, S	The main objective of the study is to demonstrate the applicability and effectiveness of the Basophil Activation Test (BAT) in detecting hypersensitivity to hymenoptera venom. In particular, the study provides a comparative evaluation of this test with skin prick tests and allergen-specific serum IgE measurements, both in terms of clinical sensitivity and positive predictive values.	This study included a total of 43 patients (General population. 14% under 10 years of age, 55.8% between 10 and 20 years of age; 30.2% over 20 years of age) who experienced systemic allergic reactions following stings from Apis mellifera and Vespula vulgaris, and who were subsequently treated with venom immunotherapy (VIT).	43 patients	The study examines the advantages and limitations of three different diagnostic methods (basophil activation test, skin prick test and specific IgE measurement) and assesses the potential contribution of BAT in the diagnosis of hymenoptera venom allergy. The study highlights the need for updated approaches for more accurate and effective detection and management of hypersensitivity conditions resulting from hymenoptera stings.	The study determined that the overall clinical sensitivities of the BAT, specific serum IgE (sIgE) and skin prick test (SPT) for Apis mellifera were 95.5%, 95.7%, and 48.4%, respectively, while for Vespula vulgaris they were 83.3%, 100%, and 33.3%. Based on these results, the ability to predict systemic reactions to bee stings is ranked as: spIgE > BAT > SPT. Furthermore, skin tests in the early stages showed a sensitivity of 67% and a specificity of 50% with a cut-off value of 1.5 mm, and a sensitivity of 33% and specificity of 83% with a cut-off of 2.5 mm.	The data suggest that, in terms of predicting systemic reactions to bee stings, specific IgE testing is the most reliable method, followed by BAT and finally skin testing. Furthermore, analysis of skin tests in the early stages showed that a cut-off value of 1.5 mm ensures good sensitivity (67%) but relatively low specificity (50%). Conversely, increasing the cut-off to 2.5 mm decreases sensitivity (33%), while specificity improves significantly (83%). This indicates that the choice of cut-off significantly influences the diagnostic performance of the skin test.	Not only pediatric population. Small sample size. Probably single-centre.	2
Vos et al. [74]	2013	CS, R, S	Evaluate whether adding the recombinant molecular component rVes v 5 to the natural Vespula venom extract used in diagnostic tests for determining specific IgE can improve the sensitivity of these tests in patients with a confirmed allergy to Vespula venom.	Adult patients (adult population) with confirmed systemic allergy to Vespula venom	308	Adding the molecular component rVes v 5 to the Vespula extract used in IgE tests significantly improves diagnostic sensitivity. This allows to find a significant proportion of allergic patients who would have tested ‘negative’ with the standard test to be identified, without compromising specificity.	The standard test (natural extract) detected specific IgE in 257 out of 308 patients (approximately 83.4%). Using molecular components (rVes v1 and rVes v5) increased diagnostic sensitivity to around 96%.	It drastically reduces the number of false negatives, ensuring greater reliability in the diagnosis of Vespula venom allergy, especially in contexts where early diagnosis is essential for the initiation of specific immunotherapy.	It does not concern the pediatric population. It is retrospective and monocentric. It analyses only one component rVes v 5. It does not provide clinical data on patients.	2

**Table A10:** Summary of secondary studies (i.e., systematic reviews, narrative reviews, meta-analyses) considered in order to gather the present evidence on hymenoptera venom allergy.

Author	Years	Systematic Review with/Without Meta-Analysis	Objectives	Population	Outcome	Results (Quantitative Elements)	Results (Description)	Pico
Tomei L. et al. [64]	2025	SR con MA	It identifies and summarizes the main risk factors for systemic reactions (SR) to hymenoptera venom in children, through a systematic review of the literature.	Pediatric population < 18 years of age, affected by hymenoptera stings who have experienced systemic reactions.	A history of previous systemic reactions and the number of stings are considered significant risk factors for the development of severe reactions. Greater exposure to a specific allergen, often linked to environmental factors and lifestyle, appears to increase the risk of sensitization and subsequent severe reactions. No conclusive data on genetic predisposition in pediatric patients have been reported; ethnicity appears to influence the risk of systemic reactions more in relation to the lifestyle of the population studied than to genetic makeup. With regard to gender, the available data are inconsistent. Asthma has been indicated in two studies as a possible risk factor for systemic reactions to hymenoptera in children. Advanced age in children is associated with an increased risk of systemic reactions. No definitive associations have emerged between eosinophilia or other atopic diseases and the risk of systemic reactions to hymenoptera venom.	Not applicable	In children, there is no evidence that gender or atopic diseases (e.g., allergic rhinitis, atopic dermatitis) are risk factors for systemic reactions to hymenoptera; only asthma has been associated with an increased risk in two studies. No significant correlations between reaction severity and diagnostic findings have emerged. Children allergic to bees are younger on average and have a higher risk of systemic reactions, although there are no statistically significant differences compared to wasp allergy.	1
Stoevesandt et al. [76]	2020	Reviews	Review of the literature on risk factors and clinical indicators associated with severe systemic reactions induced by hymenoptera stings. It seeks to identify and describe demographic, anamnestic and biological parameters that can help stratify the risk of developing severe or potentially fatal anaphylactic events in individuals sensitized to hymenoptera venom in order to improve the clinical management and prevention of such reactions in the field of allergology.	General population	Qualitative assessment of the frequency and severity of allergic reactions in relation to various factors, identifying risk factors associated with severe reactions (e.g., age, gender, comorbidities, previous stings, type of hymenoptera), identifying predictors of severe reactions for adequate risk stratification and clinical management.	Not applicable	Advanced age is associated with a significantly higher risk of developing severe anaphylactic reactions, with a higher prevalence in adults than in children. In addition, a slight male predominance in severe reactions has been observed, suggesting possible biological or exposure differences between the sexes. Cardiovascular comorbidities represent an important risk factor, especially in the presence of drug therapies with beta-blockers and ACE inhibitors, which can amplify the severity of the allergic response. Elevated baseline serum tryptase levels are identified as a predictive marker of severity, especially in patients with mast cell conditions or systemic mast cell hyperplasia. A history of previous systemic reactions is a strong prognostic indicator, suggesting a higher likelihood of recurrence with increased severity. Furthermore, the frequency and severity of reactions appear to be related to the intensity and repetition of exposure to hymenoptera venom. Wasp venom is associated with more severe reactions than bee venom. Occupational or recreational activities that increase exposure to hymenoptera contribute significantly to the overall risk of severe systemic reactions.	1
Giovannini et al. [70]	2024	Reviews	Critical and up-to-date summary of knowledge regarding hymenoptera venom allergy in children, with particular attention to epidemiology and clinical pictures, pathophysiology, diagnosis, clinical management and treatment.	Pediatric population	This recent review provides a comprehensive and in-depth overview of hymenoptera venom allergy in children. It addresses epidemiological aspects, clinical manifestations with relative pathogenesis (immunological or non-immunological) and classifies reactions into: local reactions, extensive local reactions, systemic reactions, toxic reactions and unusual reactions. The tools available to clinicians for diagnosis and patient management based on the type of reaction are analyzed in detail, as are therapeutic strategies, in particular venom immunotherapy (VIT).	Not applicable	Allergy to hymenoptera venom in children has an estimated prevalence of sensitization of around 3.7% in Italy. Allergic reactions mainly manifest as extensive local reactions (0.9–11.5%) and systemic reactions (less than 1% in Italy, up to 6.5% in other geographical areas). Severe systemic reactions are rarer in children than in adults and are associated with risk factors such as mastocytosis, cardiovascular comorbidities and the use of beta-blockers or ACE inhibitors. Diagnosis is based on a combination of medical history, skin tests, specific IgE dosage and molecular tests, while treatment includes acute therapy with adrenaline and VIT, which has been shown to be safe and effective even in children.	1, 2
Blank et al. [73]	2020	Reviews	This review analyses various endotypes of sting reactions, such as IgE-mediated allergy, asymptomatic sensitization or the simultaneous presence of venom allergy and mast cell disorders, including specific considerations for diagnosis and treatment. The clinical phenotypes of venom allergy are also described, such as differences in age of onset and disease severity, polysensitization, or patients unresponsive to therapy. Finally, biomarkers and diagnostic strategies that may reflect the patient’s immunological status and their value in guiding treatment are discussed.	General population	Diagnostic accuracy of molecular tests and immunological biomarkers. Identification of different response profiles (endotypes/phenotypes) related to severity and risk of reactions. Effectiveness and personalisation of VIT in relation to the patient’s profile.	Not applicable	It is highlighted that a more in-depth understanding and classification of endotypes and phenotypes in hymenoptera venom allergy may represent a promising approach to better understand the disease and strengthen personalized treatment strategies. Furthermore, greater knowledge of the immunological processes that occur during venom immunotherapy (VIT) could contribute to improving the management of this condition. Currently, the availability of molecular-based diagnostics has facilitated the distinction between primary allergy and cross-reactivity, thus supporting therapeutic decisions in polysensitized patients.	2
